# Development of a low-seroprevalence, αvβ6 integrin-selective virotherapy based on human adenovirus type 10

**DOI:** 10.1016/j.omto.2022.03.007

**Published:** 2022-03-16

**Authors:** Emily A. Bates, James A. Davies, Jana Váňová, Davor Nestić, Valerie S. Meniel, Sarah Koushyar, Tabitha G. Cunliffe, Rosie M. Mundy, Elise Moses, Hanni K. Uusi-Kerttula, Alexander T. Baker, David K. Cole, Dragomira Majhen, Pierre J. Rizkallah, Toby Phesse, John D. Chester, Alan L. Parker

**Affiliations:** 1 Division of Cancer and Genetics, School of Medicine, Cardiff University, Heath Park, Cardiff CF14 4XN, UK; 2 Department of Genetics and Microbiology, Faculty of Science, Charles University, Viničná 5, 128 44 Prague 2, Czech Republic; 3 Division of Molecular Biology, Ruđer Bošković Institute, Bijenička cesta 54, 10000 Zagreb, Croatia; 4 European Cancer Stem Cell Research Institute, Cardiff University, Cardiff CF24 4HQ, UK; 5 Division of Infection and Immunity, School of Medicine, Cardiff University, Heath Park, Cardiff CF14 4XN, UK; 6 Velindre Cancer Centre, Whitchurch, Cardiff CF14 2TL, UK

**Keywords:** oncolytic, virotherapy, adenovirus, targeting, αvβ6 integrin, low seroprevalence, structure

## Abstract

Oncolytic virotherapies (OV) hold immense clinical potential. OV based on human adenoviruses (HAdV) derived from HAdV with naturally low rates of pre-existing immunity will be beneficial for future clinical translation. We generated a low-seroprevalence HAdV-D10 serotype vector incorporating an αvβ6 integrin-selective peptide, A20, to target αvβ6-positive tumor cell types. HAdV-D10 has limited natural tropism. Structural and biological studies of HAdV-D10 knob protein highlighted low-affinity engagement with native adenoviral receptors CAR and sialic acid. HAdV-D10 fails to engage blood coagulation factor X, potentially eliminating “off-target” hepatic sequestration *in vivo*. We engineered an A20 peptide that selectively binds αvβ6 integrin into the DG loop of HAdV-D10 fiber knob. Assays in αvβ6+ cancer cell lines demonstrated significantly increased transduction mediated by αvβ6-targeted variants compared with controls, confirmed microscopically. HAdV-D10.A20 resisted neutralization by neutralizing HAdV-C5 sera. Systemic delivery of HAdV-D10.A20 resulted in significantly increased GFP expression in BT20 tumors. Replication-competent HAdV-D10.A20 demonstrated αvβ6 integrin-selective cell killing *in vitro* and *in vivo*. HAdV-D10 possesses characteristics of a promising virotherapy, combining low seroprevalence, weak receptor interactions, and reduced off-target uptake. Incorporation of an αvβ6 integrin-selective peptide resulted in HAdV-D10.A20, with significant potential for clinical translation.

## Graphical abstract

**Figure:**
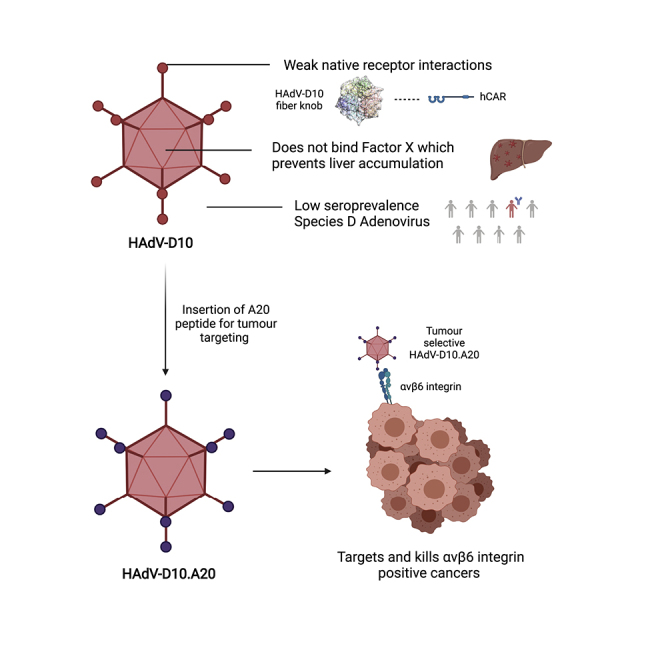

## Teaser

To date, the efficacy of adenoviral-based OV has been hampered by a lack of tumor selectivity, coupled with high rates of pre-existing anti-HAdV-C5 immunity. We engineered a low-seroprevalence adenovirus (HAdV-D10) to selectively infect αvβ6+ tumor cells. HAdV-D10.A20 has significant potential and provides an enticing platform for clinical translation.

## Introduction

Adenoviruses are widely distributed across human and animal populations. Infection by human adenovirus (HAdV) results in an array of clinical pathologies;^1^ however, the majority of infections are asymptomatic or acute and self-limiting. HAdV are non-enveloped viruses measuring 90–100 nm and arranged in an icosahedral capsid with protruding fiber proteins at the pentameric vertices. Adenoviral structure has been reviewed extensively.^2^ There are 57 canonical HAdV serotypes, divided into seven species (A–G) based on serological testing, with over 100 adenovirus serotypes isolated to date.^3^ Primary receptor binding is serotype dependent, but generally, species A, C, E, and F interact with coxsackievirus and adenovirus receptor (CAR), while species B serotypes use CD46^4^ and/or desmoglein 2 (DSG2).^5^ Species D is the largest of the adenovirus species, although many serotypes remain poorly understood.^6^ Several species D serotypes, including HAdV-D26^7^ and HAdV-D37,^8^ bind sialic acid for cell entry. The extent of this receptor usage within the species is not fully understood, although recent developments suggest potential CD46 usage via direct hexon interaction as a means of entry.^9^ Adenoviral vectors have important clinical applications ranging from viral vaccines to oncolytic viruses.^10^ Several oncolytic HAdV virotherapies have entered clinical trials, demonstrating safety and feasibility, although delivery and efficacy require optimization before they are suitable as effective cancer therapies. There is mounting excitement regarding the potential application of oncolytic viruses to prime the tumor microenvironment (TME) for immunotherapies and the prospective for novel virotherapy combinations.^11^

HAdV-C5-based vectors have been extensively evaluated and are predominantly used despite associated limitations. HAdV-C5 binds CAR, entering via αvβ3/5 integrin-mediated internalization.^12^ CAR is localized at tight junctions between cells, is expressed ubiquitously throughout the body,^13^ and is reported to be downregulated in certain cancers,^14^^,^^15^ limiting the utility of CAR as a receptor for cancer targeting. HAdV-C5 is known to interact with coagulation factor X (FX) in blood, via the hexon protein, which mediates transduction of the liver and resulting in hepatotoxicity.^16^, ^17^, ^18^ High levels of HAdV-C5 pre-existing immunity in some populations may reduce the clinical efficacy of potential HAdV-C5-based oncolytic virotherapies,^19^, ^20^, ^21^, ^22^, ^23^ where a significant proportion of the population will have previously experienced an acute adenovirus infection and developed neutralizing immunity against common HAdV serotypes.^24^^,^^25^ Activation of anti-tumor immunity, while dampening the innate host anti-viral immune response, is essential to the success of oncolytic adenovirotherapies.

Previous work in our laboratory addressed these limitations through generation of the HAdV-C5_NULL_-A20 vector.^26^ Introduction of fiber mutations, termed KO1, ablated CAR binding,^27^, ^28^, ^29^, ^30^ while the hexon was modified to prevent FX interaction, and a RGD > RGE mutation in the penton base was included to prevent binding αvβ3/5 integrins. The resulting basal Ad5_NULL_ vector was targeted to αvβ6 integrin through insertion of a 20-amino acid peptide, NAVPNLRGDLQVLAQKVART (A20), native to foot and mouth disease virus (FMDV).^31^ A20-modified viruses^32^, ^33^, ^34^, ^35^ selectively infect cells expressing αvβ6 integrin, a surface protein upregulated in several epithelial carcinomas,^36^ including breast, ovarian, pancreatic, and colorectal.^37^, ^38^, ^39^ Targeting αvβ6 integrin is advantageous in the context of cancer therapies, as it drives metastasis and tumor invasion through TGF-β activation and is consequently associated with poor prognosis.^40^, ^41^, ^42^

HAdV-D10 is a rare serotype isolated from the eyes of patients with conjunctivitis.^22^^,^^43^, ^44^, ^45^ Vogels et al. demonstrated a relatively low seroprevalence rate against HAdV-D10 of approximately 10% in a European cohort.^22^ Here, we investigate a similar approach to develop a low-seroprevalence, highly tumor-selective agent for optimal delivery to tumor-based HAdV-D10. Such an approach has potential to combine the specificity and selectivity of Ad5_NULL_-A20 with a natural ability to evade pre-existing anti-vector immunity, providing an idealized platform for successful intravenous delivery to αvβ6 integrin-expressing tumors.

## Results

### HAdV-D10 knob binds with weak affinity to known adenoviral receptors

**Figure 1:**
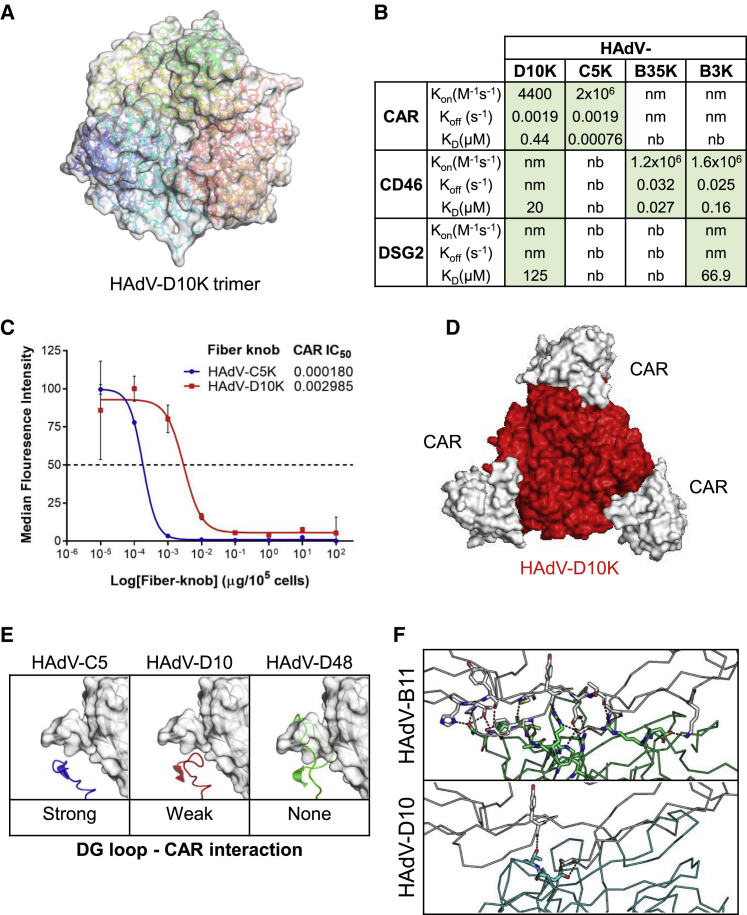
Characterization of the HAdV-D10 fiber knob and its binding receptors (A) 2.5-Å crystal structure of the HAdV-D10 fiber knob protein (HAdV-D10K). (B) Surface plasmon resonance data demonstrates HAdV-D10K binding to CAR, CD46, and DSG2 (nm, kinetics too fast to measure; nb, no binding; Green, specific binding detected). (C) Recombinant HAdV-D10K and HAdV-C5K protein binding in CHO-CAR cells using a titration of CAR-specific primary antibody and an Alexa 488-tagged secondary antibody. Data are shown as median fluorescence intensity (MFI) with SD, and IC_50_ values are shown. (D) Trimeric HAdV-D10 fiber knob (red) shown in complex with CAR receptor (white). (E) Predictive modeling of the CAR (white) and DG loop interaction of HAdV-D10 (red) compared with HAdV-C5 (blue) and HAdV-D48 (green). (F) Predictive modeling of CD46-binding sites for HAdV-D10 (cyan) and HAdV-B11 (green), a known CD46-binding adenovirus. Red dashes indicate binding potential. Structural analysis performed using Pymol.

We determined the crystal structure of HAdV-D10 fiber knob (HAdV-D10K; Figure 1A) at 2.5Å and 3.4Å (deposited as PDB entries PDB: 6ZC5 and PDB: 6QPM, respectively). Crystallization conditions, data collection, and refinement statistics are included in Table S1. Electron density maps are shown around selected parts of the structure in Figure S1. The interactions of species D adenoviruses with cellular receptors are poorly characterized. We investigated binding of HAdV-D10K to known adenoviral receptors CAR, CD46, and DSG2 by using surface plasmon resonance (Figure 1B). HAdV-D10K was able to bind to all three receptors, but the interaction was weak for CD46 and DSG2 (*K*_D_: 20 and 125 μM). The on/off rate for these receptors could not be measured, as the receptor-knob complex dissociated too quickly to measure kinetics. Binding kinetics for HAdV-C5K, HAdV-B35K, and HAdV-B3k are also shown as controls binding CAR, CD46, and DSG2, respectively (individual SPR plots shown in Figure S2). We demonstrated that HAdV-D10K forms a stronger interaction with CAR than CD46 and DSG2 (0.44 μM). This is still considered a weak interaction in comparison with CAR binding to HAdV-C5 knob (0.76nM). We investigated the ability of HAdV-D10K to interact with CAR in further detail. IC_50_ levels of recombinant HAdV-D10 knob proteins were gauged using CHO-CAR cells (Figure 1C). The data demonstrate that HAdV-D10K binds CAR with an apparent 16.5-fold lower affinity than HAdV-C5K, as indicated by the IC_50_ values. Predictive homology modeling of the trimeric HAdV-D10 knob in complex with CAR confirmed that HAdV-D10K can form a structural interface with CAR (Figure 1D); however, the extended DG loop inhibits binding to CAR, resulting in an overall lower affinity than HAdV-C5K (PDB: 6HCN) due to steric hindrance (Figure 1E). HAdV-D48K (PDB: 6FJQ) was included as a control for weak-affinity CAR interaction.^46^

We investigated whether the weak interaction between HAdV-D10K and CD46 was sufficient to result in cell attachment and infection. We modeled the interaction between CD46 and HAdV-D10K using predictive homology modeling (Figure 1F). HAdV-B11K (PDB: 3O8E) was used for comparison as a known CD46-binding adenovirus. HAdV-D10K showed far fewer potential binding sites than HAdV-B11K. Therefore, our homology model confirms the surface plasmon resonance data and that any potential interaction formed with CD46 would be weak.

### HAdV-C5/D10K pseudotype infects cells via CAR and sialic acid receptors

**Figure 2:**
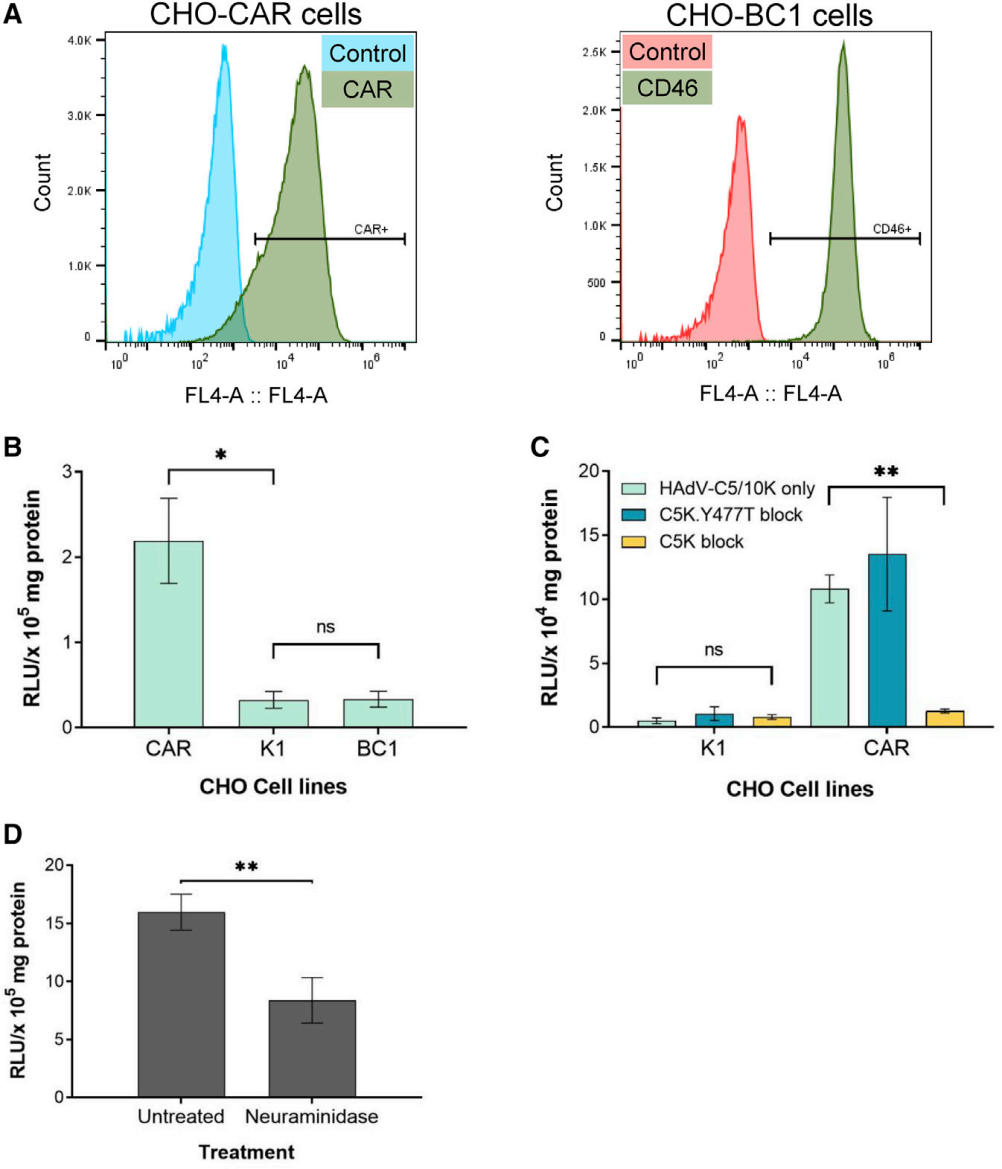
Transduction of HAdV-C5 pseudotype with HAdV-D10 fiber knob (A) Histograms demonstrating proportion of CHO-CAR cells positively stained for CAR and CHO-BC1 cells positive for CD46 determined by flow cytometry. (B) Transduction of HAdV-C5/D10K in CHO cell lines expressing no known adenoviral receptor (CHO-K1), CAR (CHO-CAR), and CD46 (CHO-BC1). Cells were infected at a viral load of 5,000 vp/cell, and luciferase production was measured at 48 h. (C) Transduction of both the HAdV-C5/D10K pseudotype in CHO-K1 and CHO-CAR cells in the presence of HAdV-C5 recombinant knob protein for CAR blocking and HAdV-C5K with a 477YT mutation that ablates CAR binding. (D) Neuraminidase assay determines HAdV-C5 pseudotype with HAdV-D10K binding to sialic acid in A549 cells. Error bars represent standard deviation. ns, p > 0.05; ∗p < 0.05, ∗∗p < 0.01, ∗∗∗p < 0.001, ∗∗∗∗p < 0.0001.

We assessed the use of these receptors in viral transduction assays. CHO-K1 cells (expressing no known HAdV receptor) and CHO-CAR and CHO-BC1 cells, expressing CAR and the BC1 isoform of CD46, respectively (Figure 2A), were infected with a pseudotyped HAdV-C5/D10K vector (Figure 2B). HAdV-C5/D10K was not able to transduce CHO-K1 cells but was able to infect CHO-CAR due to CAR binding. HAdV-C5/D10K was unable to transduce CHO-BC1, highlighting a redundancy of CD46 usage by HAdV-D10K. This agrees with both our SPR and modeling data and suggests that, although weak binding of CD46 was observed, CD46 engagement was not robust enough to result in productive infection.

After establishing that HAdV-D10K can bind CAR with low affinity, we confirmed that pseudotyped HAdV-C5/D10K could engage CAR and infect cells (Figure 2C). CHO-K1 and CHO-CAR cells were infected with HAdV-C5/D10K in the presence of CAR blocking with either HAdV-C5 recombinant knob (C5K) or HAdV-C5 recombinant knob with a Y477T CAR-binding ablating mutation (C5K.Y477T). CHO-K1 cells showed limited viral transduction due to the lack of a receptor for attachment. HAdV-C5/D10K could transduce CHO-CAR cells both without blocking and in the presence of C5K.Y477T protein, but transduction was blocked by the high-affinity C5K protein, confirming that HAdV-C5/D10K is able to bind and use CAR for cellular infection. Additionally, we performed a hemagglutination assay to assess binding of CAR on erythrocytes stimulating hemolysis. HAdV-C5 was used as a positive control for hemolysis, and HAdV-C5.KO1 with ablated CAR binding was used as a negative control. As predicted, HAdV-D10 and HAdV-C5/D10K demonstrated minimal hemolysis due to weak CAR interactions (Figure S3).

As HAdV-D10 forms only weak interactions with CAR, CD46, and DSG2 it is unlikely that they are used as primary receptors. Several species D adenoviruses have been reported as binding and using sialic acid.^8^ To investigate whether HAdV-D10K utilizes sialic acid, we infected neuraminidase-treated A549 cells with HAdV-C5/D10K (Figure 2D). Cells treated with neuraminidase demonstrated significantly decreased transduction (p < 0.05). Although this experiment was not definitive, it suggests that HAdV-C5/D10K may, like other species D adenoviruses, be able to use sialic acid to an extent for cellular entry. Further structural analysis is required to confirm whether HAdV-D10 is capable of binding sialic acid.

### HAdV-D10 vector does not use DSG2 or CD46 for cell entry

**Figure 3:**
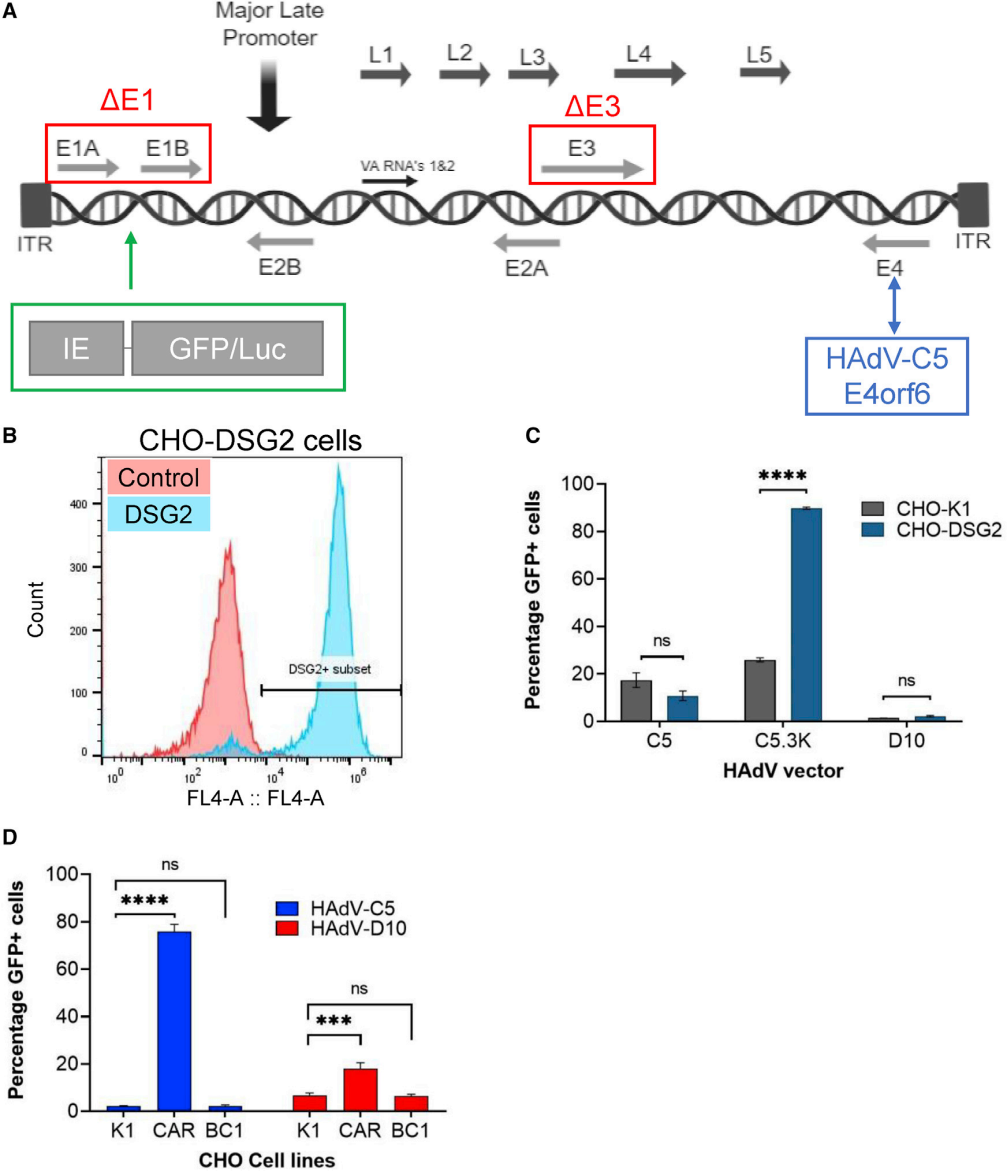
HAdV-D10 vector does not infect cells via DSG2 and CD46 (A) Schematic representing modifications made during production of HAdV-D10 vector. E1 and E3 genes were deleted, indicated by the red box; E4orf6 was replaced with HAdV-C5 E4orf6, as highlighted in blue; and green represents insertion of the transgenes GFP and luciferase under the HCMV IE promoter. (B) Histogram showing proportion of CHO-DSG2 cell line positively stained for DSG2 compared with an IgG control determined by flow cytometry. (C) Transduction of CHO-K1 and CHO-DSG2 cells by HAdV-C5 and HAdV-D10 GFP vectors, with HAdV-C5.3K used as a positive control for DSG2 receptor use. (D) Transduction of CHO-K1, CHO-BC1, and CHO-CAR cells with HadV-C5 and HAdV-D10. GFP transduction measured 72 h post-infection. Data are shown as mean of triplicate values. Error bars represent SD. ns, p > 0.05; ∗p < 0.05, ∗∗p < 0.01, ∗∗∗p < 0.001, ∗∗∗∗p < 0.0001.

To generate a HAdV-D10 vector, genomic DNA was captured within a BAC to enable rapid and efficient manipulation of the viral genome (Figure 3A). E1 and E3 were deleted, rendering the vector non-replicative, and the E4orf6 region was replaced with that of HAdV-C5 to enhance production in 293 cells.^47^, ^48^, ^49^ GFP or luciferase reporter genes were inserted under control of the human cytomegalovirus immediate early (HCMV IE) promoter.

We assessed the ability of HAdV-D10 vector to use DSG2 and CD46 receptors. A newly generated CHO cell line, referred to as CHO-DSG2, was developed in house. Flow cytometry analysis showed 93% of the population was positive for DSG2 expression compared with an IgG control (Figure 3B). CHO-K1 and CHO-DSG2 cells were infected with HAdV-C5 and HAdV-D10 vectors, as well as a HAdV-C5.3 vector, used as a positive control for DSG2 binding. No significant transduction was observed when HAdV-D10.GFP was used to transduce CHO-DSG2 compared with CHO-K1, confirming that HAdV-D10 is not able to use DSG2 as a receptor for cellular entry (Figure 3C).

A recent study suggests several species D viruses can interact with CD46 via direct engagement of the hexon.^9^ Using the whole serotype, we investigated whether HAdV-D10 can engage CD46 as an entry receptor (Figure 3D). There was no significant increase in infection of CHO cells expressing CD46 compared with CHO-K1 cells with both HAdV-C5 and HAdV-D10. Increased transduction was observed in CHO-CAR cells with both viral vectors (p < 0.0001 and < 0.001, respectively), indicating that HAdV-D10 does not engage CD46 as a cellular receptor.

### HAdV-D10 hexon does not interact with coagulation factor X

**Figure 4:**
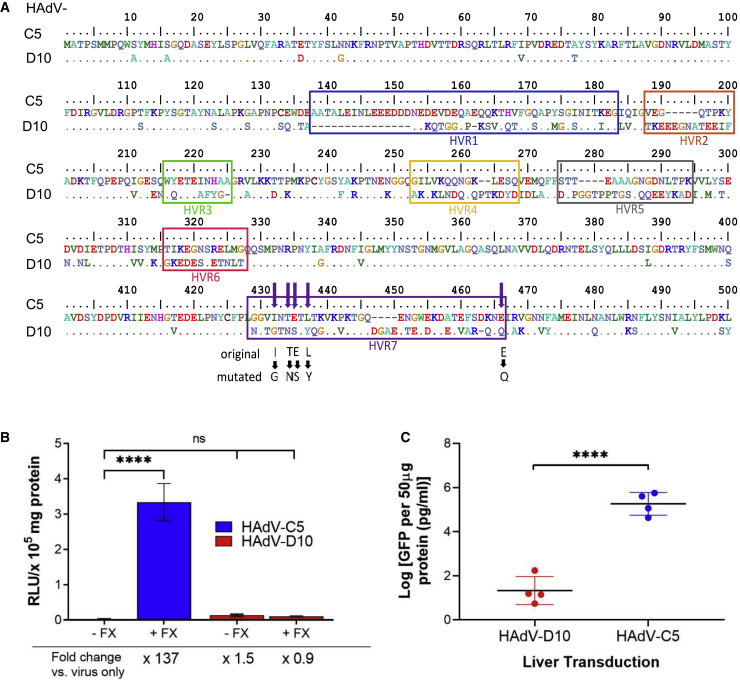
HAdV-D10 vector cannot interact with blood coagulation factor X (A) Amino acid sequence alignment of hexon hypervariable regions (HVR) in HAdV-C5 and HAdV-D10 serotypes. Sites for “HVR7” FX-binding mutation in HAdV-C5 shown in purple arrows; “original” and “mutated” amino acids involved in the point mutations are shown in bold black letters.^50^ (B) CHO-K1 cells were transduced with HAdV-C5 and HAdV-D10 vectors at 5,000 vp/cell for 3 h, and luciferase activity was measured 48 h later. (C) Liver transduction of HAdV-C5 and HAdV-D10 *in vivo*, 72 h post-intravenous injection. GFP levels were measured in 50 μg of total protein using GFP Simplestep ELISA (Abcam) and calculated from a duplicate mean, and concentration was interpolated from a standard curve and transformed using GraphPad software. Log of mean (n = 4) and standard deviation of the mean are shown. Statistical significance was determined by two-tailed unpaired t tests. ns, p > 0.05; ∗p < 0.05, ∗∗p < 0.01, ∗∗∗p < 0.001, ∗∗∗∗p < 0.0001.

Coagulation factor X (FX) is known to bind HAdV-C5 and mediate transduction to the liver, resulting in hepatotoxicity, off-target uptake, and reduced therapeutic effects of HAdV-C5-based virotherapies. Alba et al.^50^ identified and mutated FX-binding regions of HAdV-C5 hexon and key amino acids involved in this interaction through comparison with the hexon HVR7 region of HAdV-D26, known not to bind FX. To establish whether HAdV-D10 could bind FX, we aligned HAdV-C5 and HAdV-D10 hexon hypervariable regions (HVRs) highlighting key amino acids involved in FX interactions (Figure 4A). This alignment demonstrates that HAdV-D10 possesses amino acids in the HVR7 region, homologous to the mutations described to ablate FX binding. We therefore predicted that HAdV-D10 was unable to interact with FX based on the amino acid sequence and confirmed this using viral transduction assays. CHO-K1 cells were infected with HAdV-C5 or HAdV-D10 in the presence or absence of FX (Figure 4B) and compared with a virus only control. There was a 137-fold increase in expression of luciferase for HAdV-C5 in the presence of FX (p < 0.0001); however, this effect was not observed in HAdV-D10, where there was no significant difference in infection in presence of FX (0.8–fold change).

This was investigated further, *in vivo,* through biodistribution of HAdV-C5 and HAdV-D10 GFP-expressing vectors. GFP levels observed in the liver were significantly higher in mice treated with HAdV-C5 compared with HAdV-D10 (p < 0.0001) 48 h post-intravenous administration (Figure 4C). Significantly lower levels of GFP expression were also observed in the spleen in mice administered HAdV-D10 intravenously (Figure S5). These findings indicate that HAdV-D10 lacks key binding residues for FX interactions and is unable to engage and utilize FX as a means of cell entry.

### HAdV-D10 can be retargeted to αvβ6 integrin through insertion of A20 peptide

**Figure 5:**
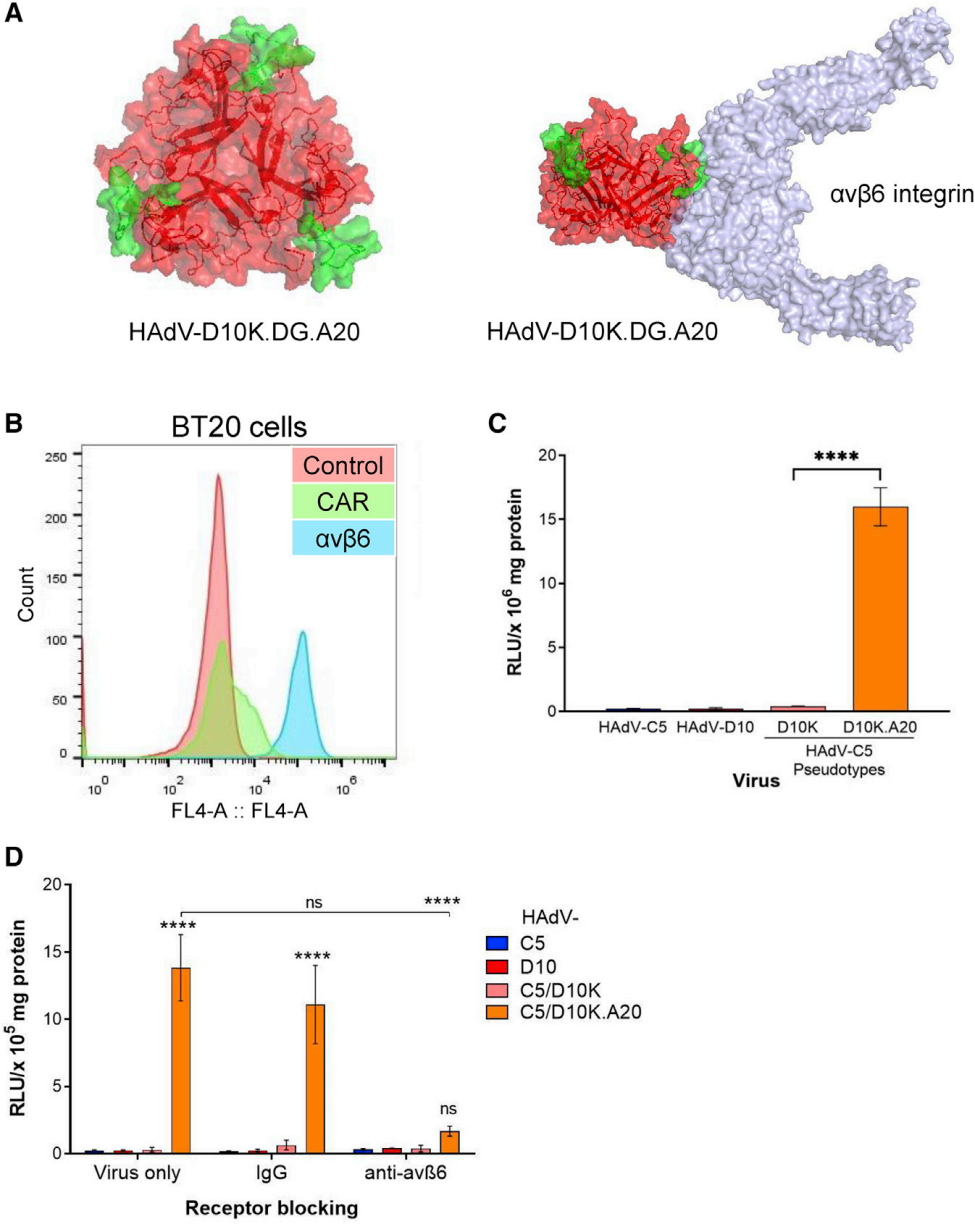
Incorporation of A20 peptide results in αvβ6 targeting (A) Predictive structural modeling of HAdV-D10 knob domains with an A20 targeting peptide insertion in DG structural loop. A20 amino acid sequence NAVPNLRGDLQVLAQKVART is highlighted in green. HAdV- D10K interaction with αvβ6 was modeled based on A20 alignment from FMDV (5NEM) using PyMol. (B) Histogram illustrating proportion of BT20 cells positive for CAR and αvβ6 cell surface receptors determined by flow cytometry. (C) BT20 cell transduction of HAdV-C5, HAdV-D10, and HAdV-C5/D10K pseudotypes. Viral infection was measured by expression of the transgene luciferase 48 h post-infection. (D) Transduction of BT20 cells in the presence of receptor-blocking antibodies. BT20 cells were preincubated with antibody (IgG and anti-αvβ6) for 30 min prior to a 1-h infection on ice. Unbound virus was removed by washing, and luciferase levels were measured 48 h post-infection. Data are plotted as a mean of n = 3, with error bars indicating standard deviation. Significance was determined using two-way ANOVA followed by Tukey’s multiple comparison test. ns, p > 0.05; ∗p < 0.05, ∗∗p < 0.01, ∗∗∗p < 0.001, ∗∗∗∗p < 0.0001.

HAdV-D10 and HAdV-C5/D10K pseudotypes were evaluated for cancer virotherapy applications. To provide a high-affinity, tumor-selective means of infection, we incorporated A20 peptide, as previously described.^26^^,^^32^, ^33^, ^34^, ^35^ A20 has high selectivity and affinity for αvβ6 integrin, which is absent on normal epithelial cells but overexpressed on aggressively transformed epithelial cells, in particular malignancies of pancreatic, breast, esophageal, and ovarian origins.^37^, ^38^, ^39^, ^40^, ^41^ We inserted A20 into the DG loop of HAdV-D10 fiber knob. Predictive models of HAdV-D10 fiber knob with A20 peptide depict the interaction with αvβ6 integrin (Figure 5A). BT20 cells were used as a model cell line for evaluating the HAdV-C5/D10K.A20 vector, as they express high αvβ6 and low CAR (Figure 5B). HAdV-C5/D10K.A20 produced significantly higher levels (p < 0.0001) of luciferase compared with HAdV-C5, HAdV-D10 and HAdV-C5/D10K (Figure 5C). Infection of HAdV-C5/D10K.A20 could be blocked using an anti-αvβ6 monoclonal antibody, thus confirming the selectivity of transduction through αvβ6 integrin (Figure 5D). These results indicate that HAdV-C5/D10K.A20 can engage and utilize αvβ6 integrin as a tumor-selective cell entry receptor through A20 peptide.

### HAdV-D10.A20 infects multiple cancer cell lines via αvβ6 integrin

**Figure 6:**
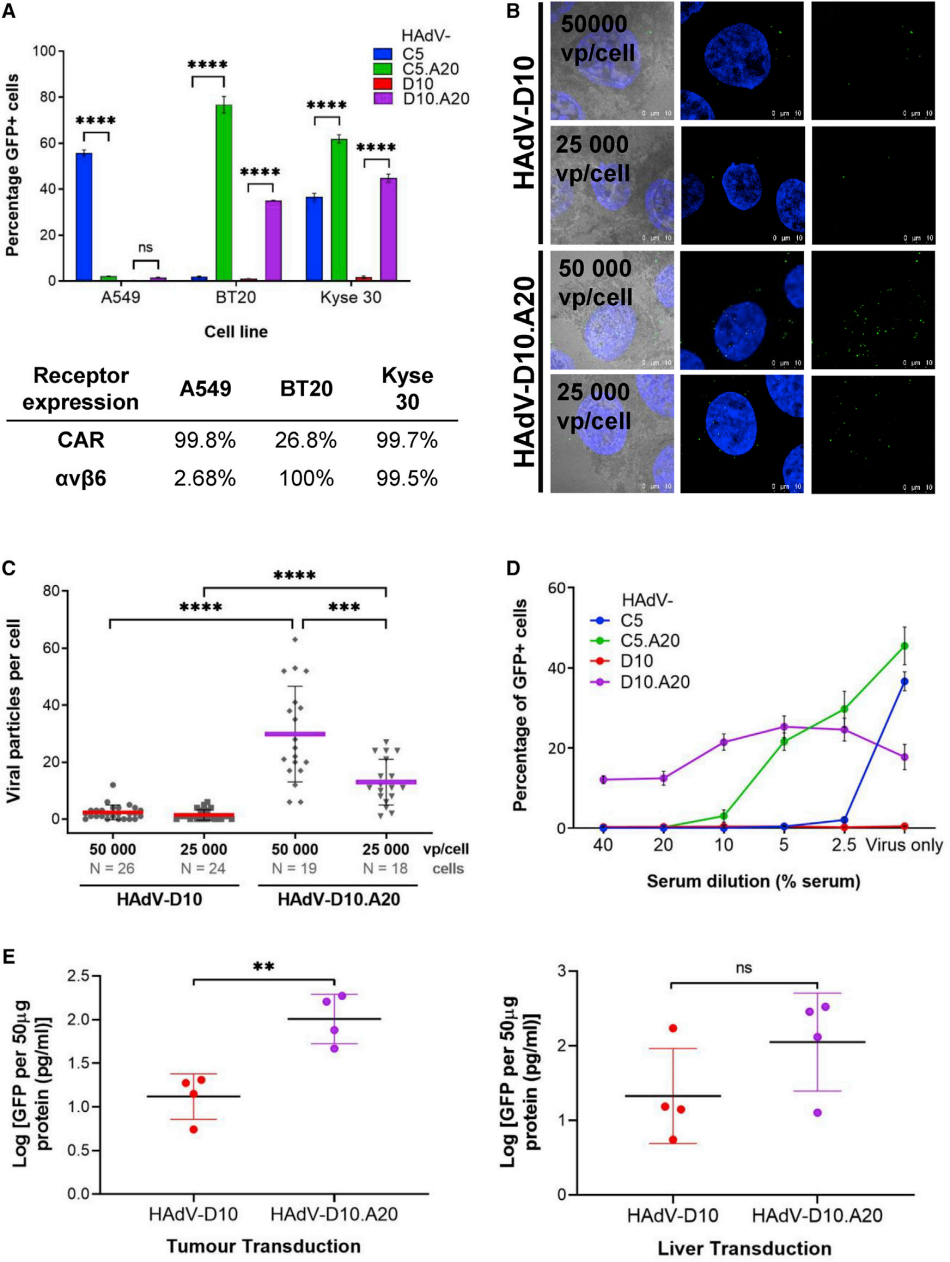
Transduction of HAdV-C5 and HAdV-D10 vectors with the A20 peptide (A) A549, BT20, and Kyse 30 cells were infected at a viral load of 10,000 vp/cell with both HAdV-C5 and HAdV-D10 vectors and the A20 modified vectors. Expression of the GFP transgene was measured using flow cytometry, 72 h post-infection. The table indicates the percentage expression of the cell surface receptors CAR and αvβ6, determined by flow cytometry. Data shown are the mean of triplicate values, with error bars representing standard error of the mean. Statistical significance was determined by two-way ANOVA using Tukey’s multiple comparisons test. (B) Microscopy assessing αvβ6 targeting of labeled HAdV-D10 vectors. Intracellular trafficking of Alexa Fluor 488-labeled HAdV-D10 and HAdV-D10.A20 in Kyse 30 cells. Green, Alexa Fluor 488-labeled HAdVs; blue, nuclei stained with DAPI; gray, reflection. The images are maximum projections of confocal stacks. Representative confocal images are shown. Scale bars, 10 μm. (C) Quantification of virus internalization efficiency, expressed as number of viral particles per cell. The horizontal bars represent means, and the error bars indicate standard deviations; the numbers of cells analyzed are indicated. (D) Viruses were pre-incubated for 15 min with serum diluted in basal medium at different concentrations (40%, 20%, 10%, 5%, 2.5%, and no serum). Kyse 30 cells were infected with 5,000 vp/cell in triplicate for each serum dilution. Expression of the GFP transgene was measured using flow cytometry 72 h post-infection. Data shown are the mean of triplicate values, with error bars representing standard deviation of the mean. (E) GFP ELISA showing biodistribution of HAdV-D10 and HAdV-D10A20 *in vivo*. Female NSG mice were inoculated subcutaneously with BT20 cells, and growth of the xenografts was monitored. GFP-expressing HAdV vectors were administered intravenously by injection into the tail vein, and tumor and liver were harvested 72 h post-infection. GFP levels were measured in 50μg of total protein using GFP Simplestep ELISA (Abcam) and calculated from a duplicate mean, and concentration was interpolated from a standard curve using GraphPad software. Data were transformed and represent log of mean (n = 4) and standard deviation of the mean. Statistical significance was determined by two-tailed unpaired t tests. ns, p > 0.05; ∗p < 0.05, ∗∗p < 0.01, ∗∗∗p < 0.001, ∗∗∗∗p < 0.0001.

We generated HAdV-D10 serotype targeting αvβ6 integrin through insertion of A20 into HAdV-D10 vector in the same position as the HAdV-C5/D10K.A20 vector. We confirmed that αvβ6-specific cell entry of HAdV-D10.A20 could be blocked by pre-incubation of BT20 cells with an anti-αvβ6 antibody (Figure S4). To determine the selectivity of these vectors, we evaluated transduction in cancer cell lines expressing varying levels of αvβ6 integrin and CAR (Figure 6A). A549, BT20, and Kyse 30 cell lines have been derived from lung carcinoma, breast carcinoma, and esophageal squamous cell carcinoma, respectively. Expressions of αvβ6 integrin and CAR were assessed by flow cytometry as indicated in Figure 6A. A549 are αvβ6 integrin low and CAR positive. Kyse 30 cells express high levels of both αvβ6 integrin and CAR. HAdV-C5.RGE.KO1.A20 (from this point referred to as HAdV-C5.A20) describes a HAdV-C5 vector containing A20 peptide in the fiber knob domain in combination with mutations in the penton base and fiber knob proteins to ablate binding to cellular integrins and CAR respectively; therefore, this virus infects only cells expressing αvβ6 integrin in the absence of FX. αvβ6 integrin-targeted viruses were unable to infect A549 cells due to lack of αvβ6 integrin. HAdV-C5.A20 readily infects both αvβ6-expressing BT20 and Kyse 30 cell lines. Interestingly, HAdV-D10 exhibits a limited infectivity in all three cell lines; however, introduction of the A20 peptide significantly increased the ability of HAdV-D10.A20 to infect both BT20 and Kyse 30 cells via αvβ6 integrin (p < 0.0001). To confirm this microscopically, HAdV-D10 and HAdV-D10.A20 labeled with Alexa Fluor 488 were used to infect Kyse-30 cells, and differences in viral internalization and trafficking were visualized (Figure 6B). The apparent increased uptake of HAdV-D10.A20 at 25,000 and 50,000 vp/cell was quantified as the number of virus particles (vp) per cell (Figure 6C). Cells infected with HAdV-D10 contained significantly fewer viral particles per cell (p < 0.0001) than HAdV-D10.A20, supporting the transduction data shown in Figure 6A.

### HAdV-D10.A20 is not neutralized in the presence of highly neutralizing anti-Ad5 serum

We investigated the effect of pre-incubation of HAdV-C5 and HAdV-D10 vectors with patient serum known to be highly neutralizing against HAdV-C5 (Figure 6D) on transduction of Kyse 30 cells. HAdV-C5 was effectively neutralized at the lowest concentration of serum (2.5%). HAdV-C5.A20 required higher concentrations of serum but could be effectively neutralized by the presence of greater than 20% serum. It was not possible to detect any effect of neutralizing serum in the case of HAdV-D10 due to the low level of infectivity at all concentrations. HAdV-D10.A20 was able to infect the Kyse 30 cells and resist neutralization even at the highest concentration of serum tested (40%). When quantified as fold change (Figure S6), this resulted in a fluctuation of fold change between 0.7 and 1.5 for HAdV-D10.A20 compared with a more than 2,000-fold decrease in infection for both HAdV-C5 (6872 fold) and HAdV-C5.A20 (2100 fold) in 40% serum. To confirm that these changes were significant, we performed an analysis comparing each serum dilution with the cell infected with virus only, plotted separately for clarity (Figure S6). HAdV-D10.A20 infection was reduced compared with virus only in the highest concentration (p = 0.05), but this was a small effect compared with that seen in the HAdV-C5-based vectors. These data suggest that HAdV-D10-based platforms can provide promising vectors that can circumvent the problems surrounding neutralization observed with HAdV-C5-based vectors.

We assessed αvβ6 targeting *in vivo*. Female NSG mice bearing subcutaneous BT20 xenografts were inoculated systemically with GFP-expressing replication-deficient HAdV vectors via intravenous injection. Tumor and liver were harvested 72 h post-infection, and transduction of GFP was determined by ELISA (Figure 6E). Some variation was observed within the individual cohorts (n = 4). There was no significant change in GFP levels between HAdV-D10 and HAdV-D10.A20 in the liver; however, there was a significant increase of GFP expression observed in the tumor mediated by HAdV-D10.A20 (p < 0.01) compared with HAdV-D10.

### Enhanced αvβ6-dependent tumor cell killing using replication-competent HAdV-D10.A20 virotherapy both *in vitro* and *in vivo*

**Figure 7:**
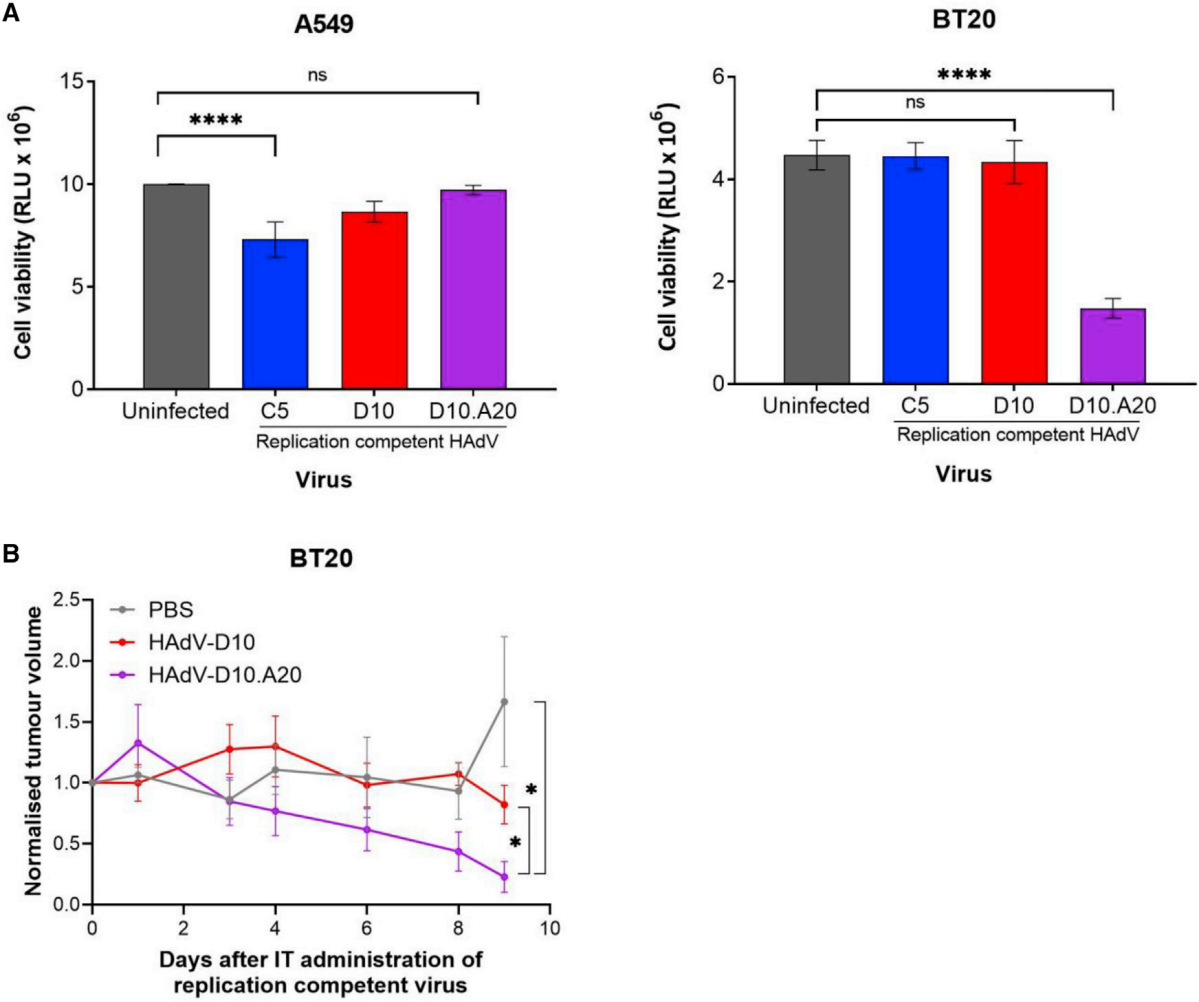
Cell viability with replication-competent HAdV-C5, HAdV-D10, and HAdV-D10.A20 *in vitro* and *in vivo* (A) A549 (αvβ6 low) and BT20 (αvβ6 high) cells were infected at a viral load of 5,000 vp/cell with replication-competent HAdV-C5 and HAdV-D10 and HAdV-D10 with the A20 modification. Cell killing was measured hours post-infection using CellTiter-Glo Luminescent Cell Viability Assay (Promega). Data shown are the mean of triplicate values with error bars representing standard deviation. Statistical significance was determined by two-way ANOVA using Tukey’s multiple comparisons test. (B) HAdV-D10 and HAdV-D10.A20 (10 × 10^10^ vp/flank) and PBS controls were administered via intratumoural (IT) injection to mice bearing BT20 xenografts. Tumor growth was measured regularly with a caliper for 9 days post-administration. Data are representative of a mean (n = 4), and significance between HAdV-D10 and HAdV-D10.A20 was determined by Mann-Whitney test. ns, p > 0.05; ∗p < 0.05, ∗∗p < 0.01, ∗∗∗p < 0.001, ∗∗∗∗p < 0.0001.

We investigated the tumoricidal activity of HAdV-D10.A20. Cell lines were infected with 5,000 vp/cell of replication-competent HAdV-C5, HAdV-D10, and HAdV-D10.A20 (Figure 7A). Cell viability was measured at 72 h post-infection. Replication-competent HAdV-D10.A20 infects BT20 cells via αvβ6 integrin, causing significant cell death (p < 0.0001). No cell killing by HAdV-D10.A20 was observed in A549 cells due to the absence of αvβ6 integrin. Replication-competent HAdV-C5 infected A549 cells via CAR, resulting in cell death (p < 0.0001); however, no significant effect was observed in the low-CAR cell line BT20. Replication-competent HAdV-D10 did not cause significant cell death in either A549 or BT20 cells at 72 h.

On the basis of the *in vitro* efficacy, we then determined whether this could be effective *in vivo*. Nude mice bearing BT20 xenografts were injected intratumorally with replication-competent virotherapies, and the effects on tumor growth was monitored (Figure 7B). Direct intratumoral administration of replication-competent HAdV-D10.A20 virotherapy resulted in a significant reduction in tumor volume after 8 days compared with HAdV-D10 and PBS. No obvious signs of unexpected toxicity or weight loss were observed in mice treated with HAdV-D10 and HAdV-D10.A20 virotherapies. Tumor sections were stained for γ-H2AX, a marker of cell death, which was observed in HAdV-D10.A20-treated tumors but not in HAdV-D10- or PBS-treated tumors (Figure S7).

## Discussion

We evaluated the suitability of HAdV-D10 for use as a virotherapy. Despite being the largest adenoviral species with the advantage of low seroprevalence, few species D serotypes have been investigated as potential oncolytic virotherapies. Literature surrounding HAdV-D10 is limited and mainly focused on its pathology.^43^^,^^44^^,^^51^ The tailoring of OV for cancer applications requires detailed knowledge of native virus-host interactions to rationally engineer viruses into optimally targeted agents for therapeutic applications. We produced a 2.5-Å crystal structure of HAdV-D10 fiber knob and modeled interactions with known viral receptors, and SPR was used to investigate binding of HAdV-D10 fiber knob with CAR, CD46, and DSG2. HAdV-D10 displayed weak but detectable binding to all three receptors. DSG2 binding is of particular interest despite having the weakest interaction, since this had previously only been described as a receptor for species BII adenoviruses. HAdV-B3, a well-described DSG2 user, also binds DSG2 with low affinity. On further investigation, we demonstrated that HAdV-D10 was not able to infect CHO-DSG2 cells, and we therefore consider that, like other species D adenoviruses, HAdV-D10 does not use DSG2 as a cellular receptor. The highest-affinity interaction was with CAR. Predictive homology was used to model HAdV-D10K in complex with CAR, and the position of the DG loop interface indicated that HAdV-D10 can bind CAR, albeit at a lower affinity than HAdV-C5. Using recombinant HAdV-D10K protein, we confirmed binding to CAR *in vitro* with a 16.5-fold lower affinity than HAdV-C5K, as indicated by the IC_50_ values and demonstrated that CAR-binding HAdV-C5 recombinant knob blocks HAdV-C5/D10K infection. We conclude that, although the binding is weak, HAdV-D10 can bind and use CAR as an entry receptor. We also investigated HAdV-D10K binding to CD46 through modeling and transduction of HAdV-C5/D10K in CD46-expressing cell lines. Although several binding sites were identified, we determined that HAdV-D10K forms weak interactions with CD46, insufficient for cell entry. Several species D adenoviruses have been reported to bind and use sialic acid as a primary entry receptor. Our studies suggest that HAdV-D10 may utilize sialic acid as a mechanism for cell entry. However, the extent of this usage was not investigated further.

HAdV-D10 vectors with E1 and E3 genes deleted and expressing GFP and luciferase were produced. Interaction between HAdV-C5 hexon HVRs and FX results in rapid and efficient HSPG-mediated hepatic accumulation of HAdV-C5 following intravascular delivery, the preferred route for targeting metastases. Alignment of HAdV-C5 and HAdV-D10 HVRs identified that key FX-binding regions were not present in HAdV-D10. FX enhanced HAdV-C5 transduction but had no impact on that mediated by HAdV-D10, and biodistribution studies showed significantly lower HAdV-D10 in the liver than HAdV-C5. We therefore conclude that HAdV-D10 does not interact with FX and that HAdV-D10-based virotherapies could bypass a major site “off-target” sequestration by the liver observed using HAdV-C5-based therapies.

A20 peptide was inserted into the DG loop of the fiber knob of HAdV-D10 pseudotypes (HAdV-C5/D10K), whole serotype HAdV-D10 vectors, and wild-type HAdV-D10. A20 has been used to retarget HAdV-C5 to αvβ6 integrin,^26^ upregulated in a number of cancer types.^37^, ^38^, ^39^ HAdV-C5/D10K.A20 pseudotype infects BT20 cells in an αvβ6 integrin-dependent manner. Furthermore, the whole HAdV-D10 serotype could be similarly re-targeted through the A20 peptide. Viruses possessing the A20 peptide demonstrated a significantly higher infection rate in αvβ6-high BT20 breast and Kyse 30 esophageal cells, thus demonstrating the potential to engineer HAdV-D10 toward cancer cell selectivity without the need for modifications to ablate native tropism. We have also demonstrated, through HAdV-C5.RGD/E.KO1.A20, that additional modifications could be incorporated to further develop the HAdV-D10.A20 vector. Ablation of CAR binding through the KO1 modification would limit any residual off-target interactions with CAR on platelets and red blood cells,^52^ while mutation of the penton RGD-to-RGE-enhanced αvβ6 selectivity may further reduce uptake in the spleen.^53^

A significant obstacle contributing to reduced efficacy of HAdV-C5-based oncolytics is the proportion of patients presenting with pre-existing immunity to HAdV-C5. Alternative rarely isolated serotypes such as HAdV-D10 may provide therapies that are effective for a broader population but also offer a valuable second line of treatment in the case of patients who acquire treatment-related immunity to HAdV-C5-based virotherapies. We evaluated the effect of neutralizing antibodies found in patient serum on viral transduction. In the presence of highly neutralizing donor serum, HAdV-C5 was rapidly neutralized at the lowest concentration (2.5% serum), and HAdV-C5.A20 was neutralized by the presence of 10% serum. HAdV-D10.A20 was not neutralized even in the presence of 40% serum, suggesting that HAdV-D10 may provide an attractive alternative to the currently used HAdV-C5-based virotherapies to circumvent pre-existing anti-adenoviral immunity.

We compared the tumor and liver uptake of GFP-expressing vectors *in vivo* following systemic application and demonstrated increased tumor-selective transduction through incorporation of A20 peptides, whereas transduction of other targeted tissues was not enhanced, indicating successful targeting of HAdV-D10.A20 to αvβ6-positive tumors following systemic administration.

Finally, we investigated the tumoricidal activity of HAdV-D10.A20 in αvβ6^low^/CAR^high^ A549 and αvβ6^high^/CAR^low^ BT20 cells. HAdV-C5 killed A549 cells via CAR but not BT20 cells. HAdV-D10 demonstrated consistently low levels of activity in both cell lines. HAdV-D10.A20 infected BT20 cells through engagement of αvβ6 integrin, resulting in significant cell death. We administered HAdV-D10 and HAdV-D10.A20 to mice bearing BT20 xenografts via intratumoral injection and observed a significant decrease in tumor volume 9 days post-administration of HAdV-D10.A20 compared with PBS and HAdV-D10. We have therefore developed a highly tumor-selective version of HAdV-D10 that is capable of cancer-specific cell killing and has shown efficacy *in vivo* without the need for additional de-targeting modifications. Future work to incorporate motifs and transgenes that mediate tumor-specific replication and cell killing such as dl24 mutation will further improve the efficacy of HAdV-D10.A20.

Taking all together, we generated the first reported structure of the HAdV-D10 fiber knob and demonstrated that HAdV-D10K can form weak interactions with several known adenoviral receptors and may use CAR and/or sialic acid for infection. HAdV-D10 does not bind FX, a feature likely to improve the pharmacokinetics of HAdV-D10-based virotherapies when delivered systemically, reducing off-target hepatic uptake. HAdV-D10 has limited infectivity in several different cancer types but represents an alluring platform into which we could engineer tumor selectivity. We generated a HAdV-D10.A20 virus that infects and kills cancer cells with upregulated αvβ6 integrin, even in the presence of highly neutralizing serum. Our findings therefore highlight that re-targeted HAdV-D10-based vectors may offer significant potential over HAdV-C5-based virotherapies, combining reduced off-target interactions with native receptors, providing a platform to engineer tropism toward high-affinity, “on-target” tumor-associated receptors and a capacity to circumvent pre-existing anti-HAdV-C5 immunity in the population. Such virotherapies, therefore, hold significant promise as platforms for successful systemic delivery of immunovirotherapies.

## Materials and methods

### Generation and crystallization of HAdV-D10 fiber knob

Recombinant HAdV-C5 and HAdV-D10 fiber knob proteins were generated, crystallized, and structurally characterized as previously described.^46^ Protein generation, structure determination, and predictive homology modeling methodology is outlined in the supplemental methods. Reflection data and final model were deposited in the Protein DataBank (PDB, www.rcsb.org) as entry PDB: 6ZC5. A low-resolution form of the structure was also determined and is deposited as entry PDB: 6QPM. Full crystallographic refinement statistics and conditions are provided (Table S1).

### Surface plasmon resonance

BIAcore 3000 was used to acquire binding analysis data. Human desmoglein-2 Fc chimera protein (DSG2), human CXADR Fc chimera protein (CAR) (R&D Systems, Minneapolis, MN, USA), and human CD46 protein (His tag; Sino biological, Bejing, China; approximately 500 RU) were coupled to the surface of a CM5 sensor chip using a slow flow rate of 10 μL/min. Measurements were performed at a flow rate of 30 μL/min in PBS (Sigma, Irvine, UK) at 25°C. HAdV-D10 fiber knob protein was purified and concentrated to 178 μM, and 5× 1:3 serial dilutions were prepared for each sample and injected over the relevant sensor chip. The equilibrium-binding constant (*K*_D_) values were calculated assuming a 1:1 interaction by plotting specific equilibrium-binding responses against protein concentrations followed by non-linear least squares fitting of the Langmuir binding equation. For single-cycle kinetic analysis, a top concentration of 200 μM HAdV-D10K was injected, followed by four serial 1:3 dilutions. *K*_D_ values were calculated assuming Langmuir binding (AB = B × ABmax/[*K*_D_ + B]), and data were analyzed using kinetic titration algorithm (BIAevaluation 3.1).

### Cell culture

Kyse 30 and A549 cells were maintained in RPMI 1640 (Sigma, New York, NY, USA). BT20 cells were grown in MEM, α modification (Gibco, Grand Island, NY, USA). CHO cells were cultured in DMEM-F-12 (Gibco). Basal medium was supplemented with 10% fetal bovine serum, heat inactivated (FBS, Gibco), 1% L-glutamine (stock 200 mM), and 2% penicillin and streptomycin (Gibco, Paisley, UK). CHO cells expressing desmoglein-2 (termed CHO-DSG2) were generated in house using the Flp-In system (Invitrogen). CHO-DSG2 cells were maintained in DMEM-F-12 supplemented with 500 μg/mL hygromycin B (Life Technologies, Paisley, UK).

### Cell surface receptor staining

A total of 100,000 cells were washed with cold FACs buffer (5% FBS in PBS) before addition of 100 μL of primary antibody. Anti-CAR (RmcB), anti-αvβ6 (Millipore, Watford, UK), and anti-DSG2 (Abcam, Cambridge, UK) were used at a concentration of 2 μg/mL. Primary antibody was removed after 1-h incubation on ice, and cells were washed twice in FACs buffer and incubated on ice for 30 min with 1:500 dilution of Alexa 647-labeled goat anti-mouse F(ab′)2 (Life Technologies, Paisley, UK). Cells were fixed using 4% paraformaldehyde and assessed by flow cytometry on an Attune NxT (Life Technologies, Carlsbad, CA, USA). Analysis was performed using FlowJo v.10 (FlowJo, LLC) by sequential gating on cell population, singlets, and Alexa 647-positive cells and plotted as median fluorescence intensity (MFI).

### IC_50_ assay using recombinant knob protein

A total of 20, 000 CHO-CAR cells^13^ per well were seeded in a V-bottomed plate and washed with PBS. All subsequent steps were conducted on ice. Serial dilutions of knob protein were made in serum-free RPMI 1640 to a concentration range of 0.0001–100 μg/10^5^ cells. Recombinant protein dilutions (in triplicate) were added to the cells for 1 h. Unbound protein was removed by washing twice, and staining for CAR receptor was carried out as above. IC_50_ curves were fitted by non-linear regression using GraphPad software to determine IC_50_ concentrations.

### Generation of viral vectors

HAdV-C5.3K was kindly gifted by Professor André Lieber, University of Washington. Pseudotyped HAdV-C5/10K and HAdV-C5/10K.A20 vectors were produced by AdZ recombineering using previously described methods.^32^^,^^54^ To generate BAC DNA containing the HAdV-D10 genome, HAdV-D10 virus was obtained from ATCC and passaged in A549 cells. A capture BAC containing 500 bp homology to each end of the HAdV-D10 genome was generated and used to capture the genome by recombination in SW102 bacteria. E1 and E3 genes were deleted, and the HAdV-D10 E4orf6 region was replaced with the HAdV-C5 E4orf6 to enhance production in 293 cells. GFP or luciferase transgenes were inserted under the control of a HCMV promoter replacing E1. HAdV-D10 was retargeted to αvβ6 insertion of the A20 peptide into the DG loop of the HAdV-D10 fiber knob, as this site is amenable to modification in the species D adenovirus.^32^ The primers used are detailed (Table S2). Viral DNA was extracted using a QIAamp MinElute Virus Spin Kit. Virus transfection and purification were conducted as previously described,^32^ with further detail in supplemental methods.

### Viral transduction assays

Cells were seeded 24 h prior to infection, and viruses were diluted to stated concentrations in serum-free medium and added to cells in triplicate for 3 h at 37°C. virus inoculum was removed and replaced with complete growth medium. Transduction was measured 48 h post-infection. Luciferase expression was detected using a Luciferase Assay System kit (#E1501; Promega UK Ltd., Southampton, UK). Protein concentration (mg/mL) was determined using a Pierce BCA Protein Assay Kit (#23227; Thermo Scientific, Loughborough, UK), and absorbance was measured at λ570 nm on an iMark Microplate Absorbance Reader (Bio-Rad, Hertfordshire, UK). GFP expression was measured by flow cytometry as described. Raw data were analyzed using FlowJo v.10, gating sequentially on cell population, singlets, and GFP-positive cells. Neuraminidase and FX transduction were carried out using luciferase-expressing vectors in the presence of 50 mU/mL neuraminidase enzyme^7^^,^^55^ from *Vibrio Cholerae* (Roche, Darmstadt, Germany) or 10 μg/mL FX (Haematologic Technologies, Cambridge Bioscience, Cambridge, UK). Cell viability was measured using CellTiter-Glo Luminescent Cell Viability Assay (Promega, Madison, WI, USA), and luminescence was read using a multimode plate reader (FLUOstar Omega; BMG Labtech, Aylesbury, UK). Serum was serially diluted by half in basal medium from 80% to 5%. Serum dilutions were added at a 1:1 ratio with basal medium containing 5,000 vp/cell, giving a final well serum concentration range of 40%–2.5%. Full details of viral transduction assays are outlined in supplemental methods.

### Confocal microscopy

Kyse 30 cells were seeded on coverslips in 24-well plates at a density of 20,000 cells per well. The following day, cells were infected with Alexa Fluor 488-labeled viruses at a concentration of 25,000 or 50,000 vp/cell^56^ and transferred to 37°C for 3 h. Cells were then fixed with 4% paraformaldehyde mounted onto slides using a drop of VECTASHIELD Antifade Mounting Media containing DAPI. Confocal microscopy was carried out using a Leica TCS SP8 X scanning microscope, and images were processed using the Leica Application Suite X (LASX).

### *In vivo* experiments

Female NSG or nude mice were subcutaneously implanted with 6 × 10^6^ BT20 cells/flank with 50% Matrigel, and xenograft growth was monitored. Virus particles (1 × 10^11^) were administered through intravenous tail vein injection to each mouse for GFP expression analysis (n = 5). Liver and tumor were harvested 72 h post-infection. Tissue was stored at −80°C. Tissue was homogenized using the TissueRuptor (Qiagen). Protein was extracted from frozen tissue as recommended for the GFP SimpleSTEP ELISA (ab171581; Abcam, Cambridge, UK), and GFP was measured according to the kit protocol. Total protein was determined using a BCA assay (Pierce), and all samples were diluted to 50 μg prior to the ELISA. Absorbance was measured at OD450 using a Cytation 5 microplate reader (Biotek, VT, USA). For wild-type analysis, 10 days after injection of the cells, HAdV-D10 and HAdV-D10.A20 (10 × 10^10^ vp/flank) and PBS controls were administered via intratumoral (IT) injection. Tumor growth was measured regularly with a caliper, ensuring that no more than 15% weight loss was observed. Mice were harvested 9 days post-administration. All animal experiments were approved by the Animal Ethics Committee, Cardiff University.

### Statistics

Analysis of raw data was performed using GraphPad unless stated otherwise. Data are shown as the mean of triplicates with standard error of the mean (SEM) or standard deviation (SD). Statistical analysis was carried out as indicated and significance determined as follows: ns, p > 0.05; ∗p < 0.05, ∗∗p < 0.01, ∗∗∗p < 0.001, and ∗∗∗∗p < 0.0001.

## Supplemental information

Document S1. Supplemental methods, Figures S1–S7, Tables S1 and S2

Document S2. Article plus supplemental information
