# Supplementary material for: Development of a low-seroprevalence, αvβ6 integrin-selective virotherapy based on human adenovirus type 10
Source: Mol Ther Oncolytics. 2022 Mar 16;25:43–56. doi: 10.1016/j.omto.2022.03.007 (PMC8971729; doi:10.1016/j.omto.2022.03.007)
Supplement: Document S1. Supplemental methods, Figures S1–S7, Tables S1 and S2 [file mmc1.pdf]

OMTO, Volume *25*

Supplemental information

Development of a low-seroprevalence, αvβ6 integrin-selective virotherapy based on human adenovirus type 10

Emily A. Bates, James A. Davies, Jana Váňová, Davor Nestić, Valerie S. Meniel, Sarah Koushyar, Tabitha G. Cunliffe, Rosie M. Mundy, Elise Moses, Hanni K. Uusi-Kerttula, Alexander T. Baker, David K. Cole, Dragomira Majhen, Pierre J. Rizkallah, Toby Phesse, John D. Chester, and Alan L. Parker

Supplemental Table 1. HAdV-D10 Fiber Knob Protein Crystal Statistics – Two Forms

**Protein**	**Ad10k - hi**	**Ad10k - lo**
**PDB Entry**	**6ZC5**	**6QPM**
**Data Collection**		
Diamond Beamline	DLS I04-1	DLS I04-1
Date	2018-10-30	2018-03-12
Wavelength	0.91587	0.91587
Crystallisation Conditions	0.2 M CaCl2, 0.1 MES, 20% w/vPEG 6000	0.2 M Na NO3, 0.1 M Bis-TrisPropane, 20.0 % PEG 3350
pH	6.0	7.5
**Crystal Data**		
a,b,c (Å)	101.357, 101.357, 326.717	183.57, 183.57, 94.88
α,β,γ (°)	90.0, 90.0, 120.0	90.0, 90.0, 120.0
Space group	P31	P 63
Resolution (Å)	2.50 – 108.91	3.395 – 79.49
Outer shell	2.50 – 2.54	3.39 – 3.58
R-merge (%)	14.7 ((276.6)	10.0 (245.5)
R-pim	6.7 (123.1)	3.1 (74.7)
R-meas (%)	16.2 (303.0)	10.4 (256.9)
CC1/2	0.996 (0.732)	0.999 (0.426)
I / σ(I)	6.7 (0.8)	15.5 (1.0)
Completeness (%)	98.7 (99.3)	99.6 (97.1)
Multiplicity	5.8 (6.0)	11.3 (11.3)
Total Measurements	741,038 (38,530)	285,257 (40,066)
Unique Reflections	128,287 (6,440)	25,265 (3,551)
Wilson B-factor($Å^2$)	57.2	118.1
**Refinement Statistics**		
Non-H Atoms	17,536	8,694
R-work reflections	121,851	23,861
R-free reflections	6,341	1,235
R-work/R-free (%)	21.9 / 25.1	20.3 / 23.4
[1]Twin Law 1 / Fraction 1	H, K, L / 0.253	n/a
[1]Twin Law 2 / Fraction 2	K, H, -L / 0.254	n/a
[1]Twin Law 3 / Fraction 3	-H, -K, L / 0.245	n/a
[1]Twin Law 4 / Fraction 4	-k, -H, -L / 0.248	n/a
**[2]rms deviations**		
Bond lengths (Å)	0.013	0.008
Bond Angles (°)	1.576	1.753
[3]Coordinate error	0.050	0.480
Mean B value ($Å^2$)	78.7	172.4
**Ramachandran Statistics**		
Favoured/allowed/Outliers	1886 / 271 / 30	982 / 99 / 5
%	86.2 / 12.4 / 1.4	90.4 / 9.1 / 0.5

* One crystal was used for determining the structure.
* Figures in brackets refer to outer resolution shell, where applicable.
[1]Twin laws determined and fractions estimated automatically by REFMAC5
[2]Figures in brackets are rms targets
[3]Coordinate Estimated Standard Uncertainty in (Å), calculated based on maximum likelihood statistics.

Supplementary Table 2. Primers used in generation of viral vectors

Primer	Sequence (5'-3')
Cass in E4Orf6 Ad10 F	CGGTGATTGAGATGAAGCCGTCCTCTGAAAAGTCATCCAAGCGAGCCTCACAGTCCAAGGCCTGTGACGGAAGATCACTTCG
Cass in E4Orf6 Ad10 R	GTTCAAGGGCCCATTTCTGCTGGCAGAAGTACGACAAGGTACGCAAGAGAATCCACTACACTGAGGTTCTTATGGCTCTTG
Ad5 E4Orf6 Ad10 arms F	CGGTGATTGAGATGAAGCCGTCCTCTGAAAAGTCATCCAAGCGAGCCTCACAGTCCAAGGCTACATGGGGGTAGAGTCATAATCG
Ad5 E4Orf6 Ad10 arms R	GTTCAAGGGCCCATTTCTGCTGGCAGAAGTACGACAAGGTACGCAAGAGAATCCACTACAATGACTACGTCCGGCGTTCCATTTG
Cass in E3 Ad10 F	TGGTCAGGTTCTTCACCCAGCAACCCTTCCTGGTCGAGCGGGACCGGGGCGCCACCACCTACACCGTCTACCTGTGACGGAAGATCACTTCG
Cass in E3 Ad10 R	GGTTTGATTGGTTTCTGGGCTTTAATCAACATCAGTTCATGGGCAGGAGGTCGCGGAGTCCGCAAAGGGTCTGAGGTTCTTATGGCTCTTG
Ad10 E3 delete oligo	CAACCCTTCCTGGTCGAGCGGGACCGGGGCGCCACCACCTACACCGTCTAACCCTTTGCGGACTCCGCGACCTCCTGCCCATGAACTGATGTTGATTAAA
CMV/polyA in E1 F	GTCAAGAGGCCACTCTTGAGTGCCAGCGAGTAGAGATTTCTCTGAGCTCCGCTCCCAGAGACCGAGAAAACCTGTGACGGAAGATCACTTCG
CMV/polyA in E1 R	AAAAAGACCCTCGTAAGACACCCGCCTTTATAGTCACCTTAGCCACGCCCACTACTCACTCGACCTACCTCTGAGGTTCTTATGGCTCTTG
RPSL/sacB kn10.DG F	TTATGAAAAAGCAATTGGTTTTATGCCTAATTTGGTAGCGTATCCGAAACCCAGTAATTCTAAAAAATATCCTGTGACGGAAGATCACTTCG
RPSL/sacB kn10.DG R	TAGTTTTAATGACTGCTGGCTGATCAGGTTTTCCACCAAGATATATAGTTCCATAAACTATGTCTCTTGCCTGAGGTTCTTATGGCTCTTG
kn10.DG.A20 F	TTATGAAAAAGCAATTGGTTTTATGCCTAATTTGGTAGCGTATCCGAAACCCAGTAATTCTAAAAAATATAATGCTGTGCCCAACTTGAGAGGTGAC
kn10.DG.A20 R	TAGTTTTAATGACTGCTGGCTGATCAGGTTTTCCACCAAGATATATAGTTCCATAAACTATGTCTCTTGC CGTCCGTGCCACCTTTTGAGCCAAC
PCR HAdV-D10 knob *F*	AACACCAGACACTTCTCCAAACTGCAC
PCR HAdV-D10 knob *R*	TACACTGTGAAATGGGCTGGTGGTGG
Seq HAdV-D10 knob *F*	ATTGCTCAGGATAAGGACTCTAAACTAACTC
Seq HAdV-D10 knob *R*	AGACTGACTACCCGTGCTGGTGTAAAAATC

Supplemental Figure 1. Electron density around selected parts of the structure of HAdV-D10K. Observed density is blue at 1Å contour level, positive difference is green at +3 Å, negative difference is red at −3 Å.

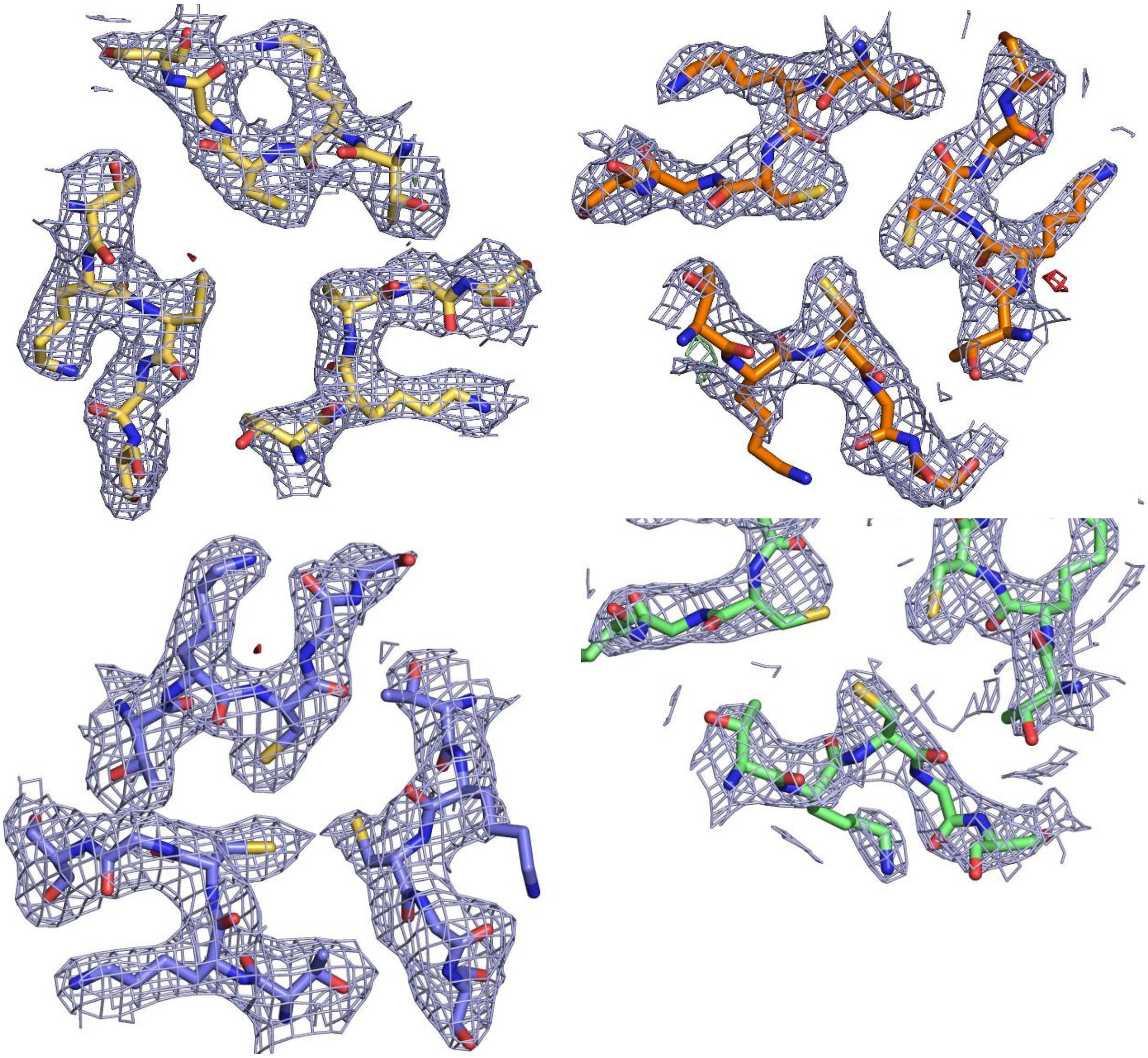

Supplemental Figure 2. SPR analysis of fiber knob protein binding. CD46, CAR and DSG2 binding to HAdV-D10K, HAdV-C5K, HAdV-B35K and HAdV-B3K fiber knob proteins was measured by SPR. The equilibrium binding constant (K_D) values were calculated assuming a 1:1 interaction by plotting specific equilibrium-binding responses against protein concentrations followed by non-linear least squares fitting of the Langmuir binding equation. For single cycle kinetic analysis, a top concentration of 200μM HAdV-D10K was injected, followed by four serial 1:3 dilutions. K_D values were calculated assuming Langmuir binding ($AB = B \times ABmax/(K_D + B)$), and data were analysed using kinetic titration algorithm (BIAevaluationTM 3.1).

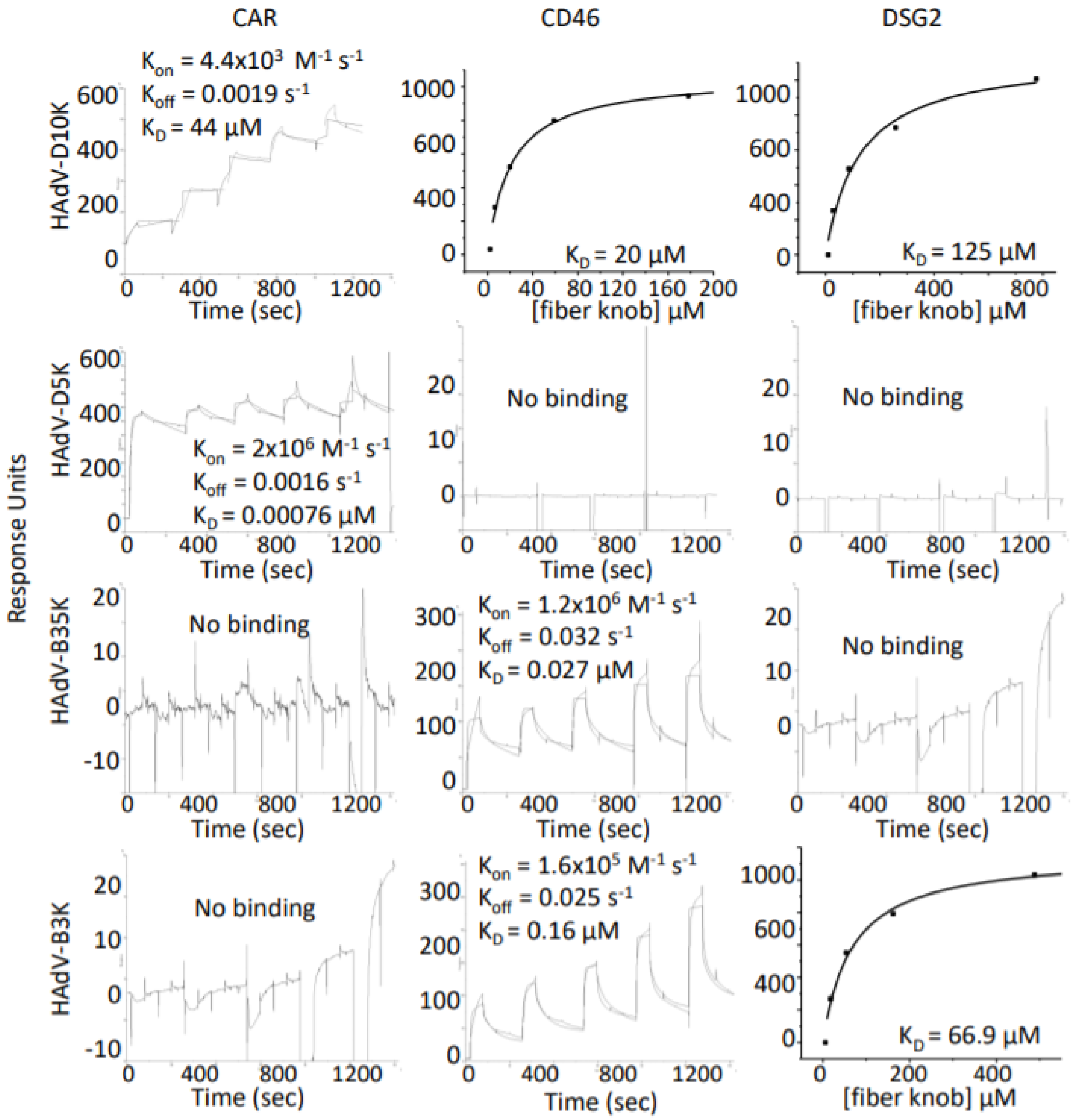

Supplemental Figure 3. Haemagglutination assay to demonstrate CAR binding through haemolysis. HAdV-C5, HAdV-C5.KO1, HAdV-D10 and HAdV-C5/D10K were combined with 1% erythrocyte solution in PBS at a concentration of $2.5x10^8$ virus particles.

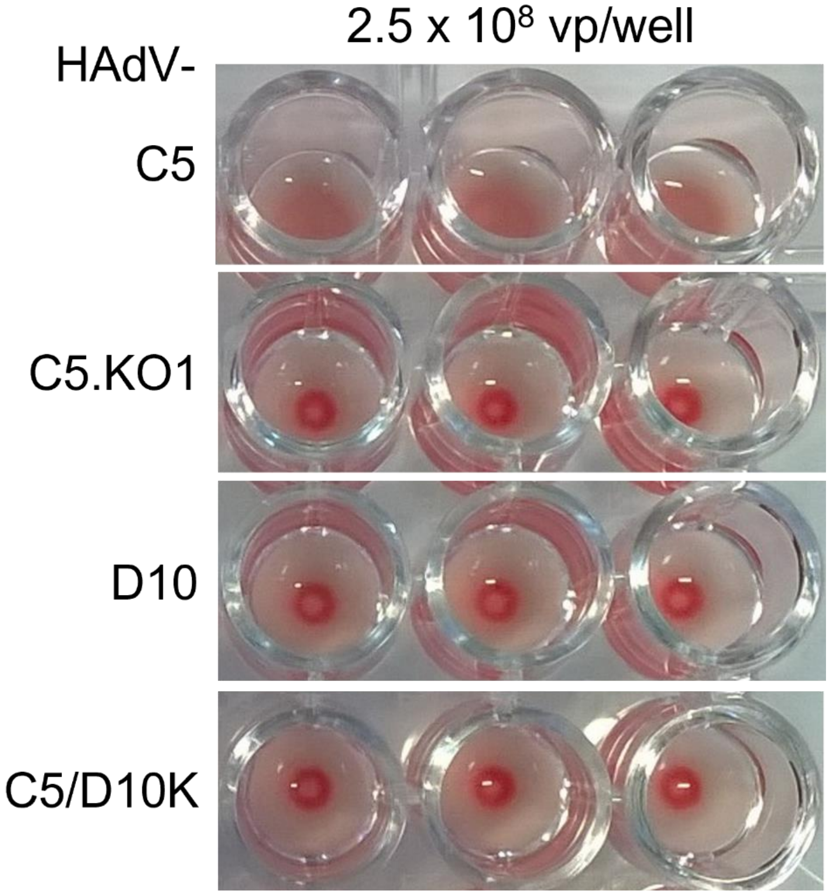

Supplemental Figure 4. Transduction of BT20 cells in the presence of receptor blocking antibodies. BT20 cells were preincubated with anti-αvβ6 antibody for 30 minutes prior to a 1-hour infection on ice, cells were then incubated for 48 hours at 37 °C. Data represents triplicate mean and error bars indicate standard deviation. Significance was determined unpaired t-test. ns, $p > 0.05$; *, $p < 0.05$; **, $p < 0.01$; ***, $p < 0.001$; ****, $p < 0.0001$.

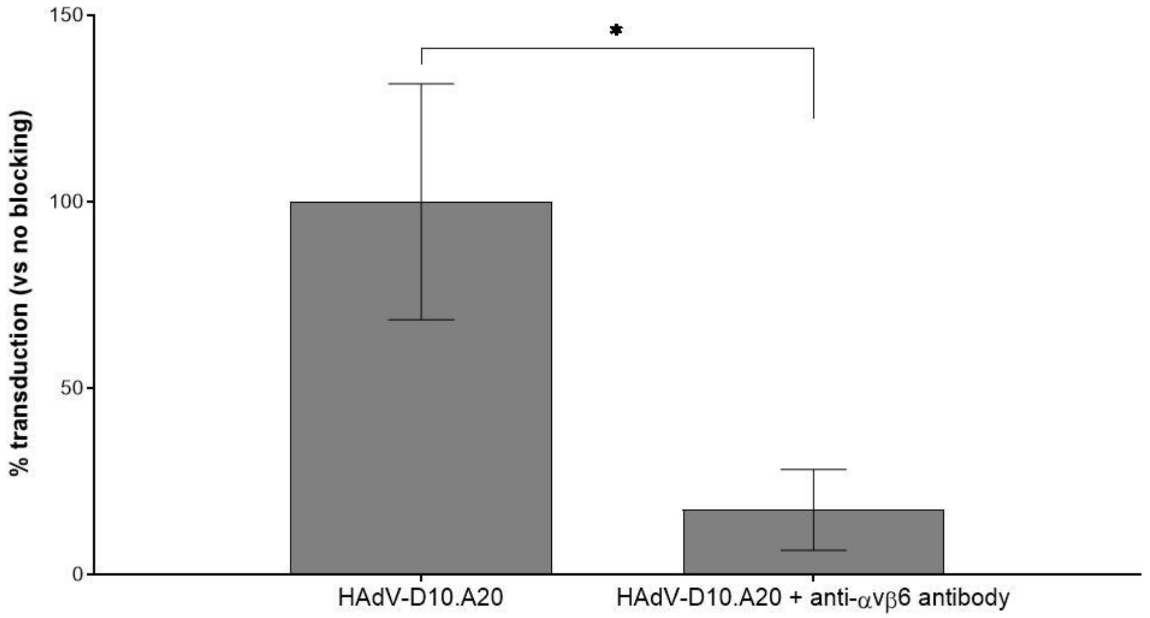

Supplemental Figure 5: Transduction of HAdV-C5 and HAdV-D10 in the spleen, 72 hours post intravenous injection. GFP levels were measured in 50µg of total protein using GFP Simplestep ELISA (Abcam) and calculated from a duplicate mean and concentration was interpolated from a standard curve and transformed using GraphPad software. Log of mean (n=4) and standard deviation of the mean have been shown. Statistical significance was determined by two-tailed unpaired t tests. ns, $p > 0.05$; *, $p < 0.05$; **, $p < 0.01$; ***, $p < 0.001$; ****, $p < 0.0001$.

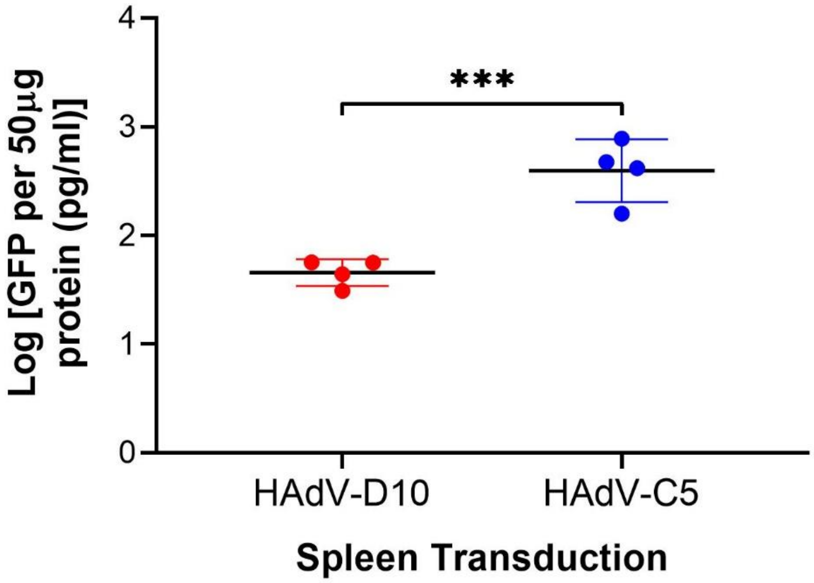

Supplemental Figure 6. Individual graphs highlighting statistical differences in Figure 6D. The table indicates fold change when compared to virus infection with no serum for each serum dilution. Data shown is a mean of triplicate values with error bars representing standard deviation of the mean. Statistical significance was determined by a One-way ANOVA using Dunnett's multiple comparisons test. Statistics indicatedwhere significantly different from virus only. ns, $p > 0.05$; *, $p < 0.05$; **, $p < 0.01$; ***, $p < 0.001$; ****, $p < 0.0001$.

**Fold change**	**40%**	**20%**	**10%**	**5%**	**2.5%**
**HAdV-C5**	**6871.9**	**2243.9**	**1427.9**	**90.1**	**17.8**
**HAdV-D10**	**1.6**	**1.5**	**1.1**	**1.1**	**2.1**
**HAdV-C5.RGE.KO1.A20**	**2100.0**	**221.6**	**15.1**	**2.1**	**1.5**
**HAdV-D10.A20**	**1.5**	**1.4**	**0.8**	**0.7**	**0.7**

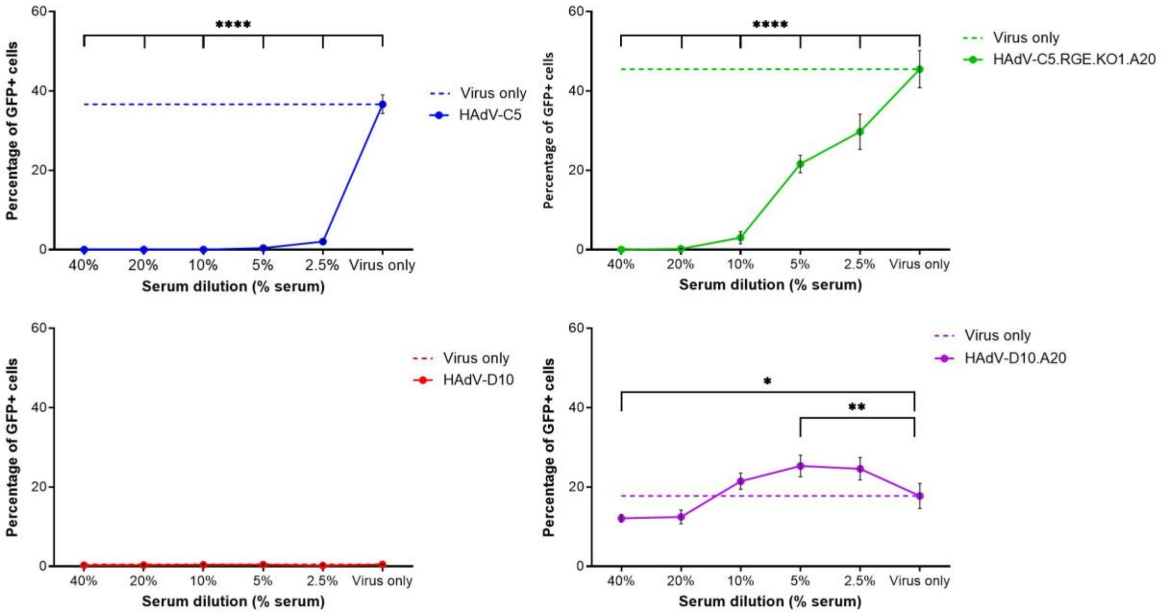

Supplemental Figure 7: BT20 tumour sections from mice treated intratumorally with PBS, HAdV-D10 and HAdV-D10.A20 were stained for gamma H2AX as a marker of cell death.

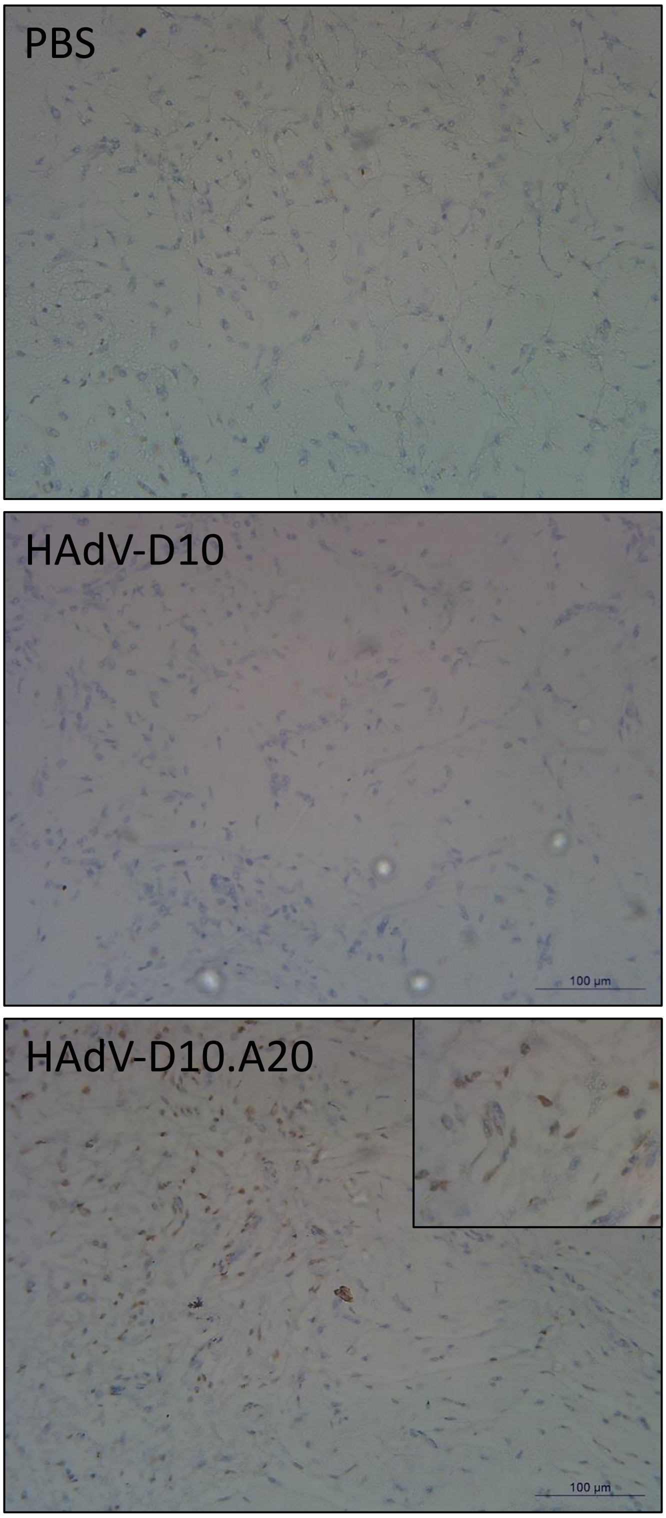

Supplemental Methods

Generation of recombinant fiber knob protein

Recombinant HAdV-C5 and HAdV-D10 fiber knob proteins were generated in SG13009 *Escherichia coli* harbouring pREP-4 plasmid and a pQE-30 expression vector containing the relevant fiber knob encoding DNA sequence. Glycerol stocks were used to inoculate 20 mL LB broth containing 100 µg/mL ampicillin and 50 µg/mL kanamycin and cultured overnight. The overnight culture was added to 1 L of TB (Terrific Broth, modified, Sigma-Aldrich) supplemented with 100 µg/mL ampicillin, 50 µg/mL kanamycin, and 8 mL of glycerol (0.8%). Cultures were placed in a shaking incubator (250 rpm) at 37 °C until they measured OD600 = 0.6. Expression of recombinant fiber knob from pQE-30 was induced by addition of IPTG to a final volume of 0.5 mM prior to incubation for 4 hours at 37 °C. Bacteria were harvested by centrifugation at 4000 g for 10 minutes at 4°C. Pellets were stored at −80°C prior to purification. Thawed pellets were resuspended in lysis buffer (50 mM Tris [pH 8.0] 300 mM NaCl, 1% (v/v) NP40, 1 mg/mL Lysozyme, 1 mM β-mercaptoethanol) and incubated at room temperature for 30 minutes, agitating to aid lysis. Lysates were clarified by a 30-minute centrifugation at 30,000 g and filtered using a 0.22 µm syringe filter (Millipore, Abingdon, UK). Purification was conducted using the AKTA FPLC. Filtered lysates were passed through a 5 mL HisTrap FF nickel affinity chromatography column (GE life sciences, 17525501) at 2.0 mL/min and washed with 5 column volumes into elution buffer A (50 mM Tris [pH 8.0], 300 mM NaCl, 1 mM β-mercaptoethanol). Protein was eluted by 30 min gradient elution from buffer A to B (buffer A + 400 mM Imidazole). Fractions were analysed by reducing SDS-PAGE, and fractions containing fiber knob were pooled and further purified using a superdex 200 10/300 size exclusion chromatography column (GE 10/300 GL, GE Life Sciences) in crystalisation buffer (10 mM Tris, [pH 8.0] and 30 mM NaCl). Fractions correlating to area under the chromatogram peaks were collected and analysed by SDS-PAGE. Pure fractions were concentrated by centrifugation in Vivaspin 10,000 MWCO (Sartorius, Goettingen, Germany) preceding crystalisation.

Crystallisation of HAdV-D10 fiber knob (HAdV-D10k) and structure determination

Crystallisation experiments were set up in a 96-well sitting-drop plate. PACT Premier commercial screen from Molecular Dimensions (UK), was used to equilibrate 200 nL drops of protein against 60 mL drops of screen. The best crystals appeared in the condition 0.2 M $CaCl_2$, 0.1 MES, 20% w/v PEG 6000, pH 6.0. Crystals were harvested into thin plastic loops, cryocooled and transferred to the Diamond Synchrotron Light Source at Harwell, UK. Data collection was conducted at DLS Beamline I04-1. Structure determination of HAdV-D10k was conducted according to previously described methods (50). Reflection data and final model were deposited in the Protein Data Bank (PDB, www.rcsb.org) as entry 6ZC5. A low-resolution form of the structure was also determined and is deposited as entry 6QPM. Full crystallographic refinement statistics and conditions are given in Supplementary Table 1.

Predictive homology modelling

Fiber knob proteins were modelled in complex with CAR or CD46 using the template of existing HAdV-C5K (PDB 6HCN) for CAR binding or the HAdV-B11K (PDB 3O8E) for CD46 binding structures. Non-protein components and hydrogens were deleted from the template model and the fiber knob protein of interest. The Cα chains of each fiber knob protein were aligned in such a way as to achieve the lowest possible RMSD. Models containing only the HAdV-D10k fiber knob protein and the ligand were saved, and energy minimization was performed, using the YASARA self-parametrising energy minimisation algorithm via the YASARA energy minimisation server (www.YASARA.org). Results were visualised and adapted for publication using PyMoL visualisation software.

Virus preparation and purification

DNA was amplified using a maxiprep kit as described in the manufacturer's instructions (Nucleobond BAC 100, Macherey-Nagel). DNA concentration was determined using a Nanodrop ND-1000 (Thermo Scientific, UK). Virus particles were generated by lipofectamine transfection onto a T25 CELLBIND flask of T-REX cells or 293β6 cells. Cells were collected when cytopathic effect (CPE) was apparent, and virus was amplified using the respective cell lines. Caesium chloride (CsCl) two-step purification method was used to extract pure virus. Alexa-Fluor 488 labelled viruses were prepared using CsCl purification with dialysis into PBS. Viral particles were then incubated for two hours at RT with 20-fold excess of Alexa-Fluor488-TFP (Molecular Probes). Zeba Spin desalting columns (Pierce) were used to purify labelled viral particles. Viral titer was determined using both microBCA and NanoSight (Malvern Panalytical) technology. Viruses were maintained at −80°C for long term storage.

Neuraminidase assay

A549 cells were seeded at a density of 20,000 cells per well and allowed to adhere overnight. Cells were washed twice with PBS before addition of 50 µL of neuraminidase enzyme from *Vibrio Cholerae* (11080725001, Roche) used at 50 mU/mL. Cells were incubated at 37°C for 1 hour prior to washing with cold PBS. Viral transduction was carried out as described above using luciferase system on ice to ensure cleaved sialic acid is not replenished.

FX transduction

For assessment of the impact of physiological concentrations of human coagulation FX on transduction efficiency, viral transduction was performed as described for the luciferase assay above. Virus dilutions were prepared in serum-free medium that was supplemented with 10 µg/mL of FX (#HCX0050, Haematologic Technologies, Cambridge Bioscience, Cambridge, UK) for 3 h.

Viral transduction in presence of serum

To determine the effect of neutralising antibodies on viral transduction, serum was collected from the blood of a healthy donor. Serum was serially diluted by half in basal media from 80% to 5%. Serum dilutions were added at a 1:1 ratio with basal media containing 5000 vp/cell giving a final well serum concentration range of 40% to 2.5%. GFP expression was measured by flow cytometry as described.

Cell viability assay

Cells were seeded at a density of 10,000 cells per well in triplicate in a white, opaque bottom 96 well plate (Corning™ 3915). Cells were seeded 24 hours prior to viral infection. Wildtype HAdV-C5, HAdV-D10 and HAdV-D10.A20 were added to cells at a concentration of 5,000 vp/cell. The plates were incubated at 37°C and the viability was measured using CellTiter-Glo® Luminescent Cell Viability Assay (Promega). CellTiter-Glo reagents were prepared according to the manual and 50 µL of the reagent was added to the cells. The plates were protected from light and shaken to fully lyse cells before luminescence was read using a multimode plate reader (FLUOstar Omega, BMG Labtech, Aylesbury, UK).

Haemagglutination assay

CAR tropism was assessed in standard haemagglutination assay. Fresh blood was obtained under ethics permission for collection of blood from healthy volunteers (Cardiff University School of Medicine Research Ethics committee (SMREC) reference number: 16/45). Erythrocytes were pelleted and transferred to a fresh 15-mL tube then washed two times with 5 mL of PBS by centrifugation for 8 min at 1000rpm. The erythrocytes were then diluted to 1 % (v/v) in PBS, and virus dilutions were prepared at 2.5 x 108 in PBS. 50 µL of erythrocyte suspension was added in each well of V-bottom 96-well plate, 50 µL of virus dilutions added on the top and incubated for 2 hours.

Immunohistochemistry

Tumours sections from mice treated with PBS, HAdV-D10 and HAdV-D10.A20 were mounted on slides were treated in Xylene and graded ethanol to remove the paraformaldehyde. Antigen retrieval was carried out in citrate buffer pH 6. 15) in a pressure cooker. Once cool, anti-γH2AX (Novus Biologicals, NB100-2280) was added at a concentration of 1/300 in 2% BSA /TBS/Tween and left at 4 °C overnight. Primary was removed and biotinylated goat anti-rabbit secondary antibody (BA-1000 Vectorlabs) was added at 1/200 for 30 minutes at room temperature. Slides were then stained using ABC kit Vectorlabs for 30 minutes and mounted with cover slips before imaging.
